# Dysfunction of the CNS-Heart Axis in Mouse Models of Huntington's Disease

**DOI:** 10.1371/journal.pgen.1004550

**Published:** 2014-08-07

**Authors:** Michal Mielcarek, Linda Inuabasi, Marie K. Bondulich, Thomas Muller, Georgina F. Osborne, Sophie A. Franklin, Donna L. Smith, Andreas Neueder, Jim Rosinski, Ivan Rattray, Andrea Protti, Gillian P. Bates

**Affiliations:** 1 Department of Medical and Molecular Genetics, King's College London, London, United Kingdom; 2 CHDI Management Inc./CHDI Foundation Inc., Los Angeles, California, United States of America; 3 King's College London British Heart Foundation Centre of Excellence, Cardiovascular Division and Division of Imaging Sciences and Biomedical Engineering, King's College London, London, United Kingdom; University of Minnesota, United States of America

## Abstract

Cardiac remodelling and contractile dysfunction occur during both acute and chronic disease processes including the accumulation of insoluble aggregates of misfolded amyloid proteins that are typical features of Alzheimer's, Parkinson's and Huntington's disease (HD). While HD has been described mainly as a neurological disease, multiple epidemiological studies have shown that HD patients exhibit a high incidence of cardiovascular events leading to heart failure, and that this is the second highest cause of death. Given that huntingtin is ubiquitously expressed, cardiomyocytes may be at risk of an HD-related dysfunction. In mice, the forced expression of an expanded polyQ repeat under the control of a cardiac specific promoter led to severe heart failure followed by reduced lifespan. However the mechanism leading to cardiac dysfunction in the clinical and pre-clinical HD settings remains unknown. To unravel this mechanism, we employed the R6/2 transgenic and *Hdh*Q150 knock-in mouse models of HD. We found that pre-symptomatic animals developed connexin-43 relocation and a significant deregulation of hypertrophic markers and *Bdnf* transcripts. In the symptomatic animals, pronounced functional changes were visualised by cardiac MRI revealing a contractile dysfunction, which might be a part of dilatated cardiomyopathy (DCM). This was accompanied by the re-expression of foetal genes, apoptotic cardiomyocyte loss and a moderate degree of interstitial fibrosis. To our surprise, we could identify neither mutant HTT aggregates in cardiac tissue nor a HD-specific transcriptional dysregulation, even at the end stage of disease. We postulate that the HD-related cardiomyopathy is caused by altered central autonomic pathways although the pathogenic effects of mutant HTT acting intrinsically in the heart may also be a contributing factor.

## Author Summary

Huntington's disease (HD) is a neurodegenerative disorder for which the mutation results in an extra-long tract of glutamines that causes the huntingtin protein to aggregate. It is characterized by neurological symptoms and brain pathology that is associated with nuclear and cytoplasmic aggregates and with transcriptional dysregulation. Despite the fact that HD has been recognized principally as a neurological disease, there are multiple epidemiological studies showing that HD patients exhibit a high rate of cardiovascular events leading to heart failure. To unravel the cause of cardiac dysfunction in HD models, we employed a wide range of molecular and physiological methods using two well established genetic mouse models of this disease. We found that pre-symptomatic animals developed aberrant gap junction channel expression and a significant deregulation of hypertrophic markers that may predispose them to arrhythmia and an overall change in cardiac function. These changes were accompanied by the re-expression of foetal genes, apoptotic cardiomyocyte loss and a moderate degree of interstitial fibrosis in the symptomatic animals. Surprisingly, we could identify neither mutant HTT aggregates in cardiac tissue nor a HD-specific transcriptional dysregulation. Therefore, we conclude that the HD-related cardiomyopathy could be driven by altered central autonomic pathways.

## Introduction

Huntington's disease (HD) is an inherited neurodegenerative disorder caused by the expansion of a polyglutamine (polyQ) stretch within the huntingtin protein (HTT). It is characterized by neurological symptoms and brain pathology, particularly neurodegeneration in the basal ganglia and cerebral cortex [1]. In mammals, HTT is expressed in many tissues and organs [2], [3]. Its subcellular localization is very dynamic and it has been found to colocalize with organelles such as the nucleus, endoplasmic reticulum, Golgi apparatus and endosomes [4]. HTT is predicted to form an elongated superhelical solenoid structure due to a large number of HEAT motifs suggesting that it plays a scaffolding role for protein complex formation [5]. More than 200 HTT interaction partners have been identified; these can be classified based on their function and include proteins that are involved in gene transcription, intracellular signalling, trafficking, endocytosis, and metabolism [6]. The process of mutant HTT self-aggregation is an early event in HD progression which may lead to the pathological features of HD. Insoluble polyQ aggregates, a hallmark of HD pathology, are detectable at the presymptomatic stage in HD *post mortem* brain [7] and can be found in both the brain as well as in many non-central nervous system tissues in HD mouse models [8], [9]. There is growing evidence to indicate that peripheral pathologies such as weight loss and skeletal muscle atrophy may not be a consequence of neurological dysfunction or neurodegeneration and might make a significant contribution to the disease presentation and progression [10]. Therefore it is important to identify the peripheral abnormalities that may contribute to disease progression and might present targets for new treatment strategies.

To date, our knowledge of heart pathology in HD is very limited. However, multiple epidemiological studies have shown that heart disease is the second cause of death in patients with HD and the following frequencies have been reported: 12.2% [11], 19% [12] and 24.4% [13]. Moreover, clinical studies have revealed that HD patients have enhanced cardiovagal activity [14], reduced cortical and subcortical blood flow [15] and a reduced heart rate [16]. A further study showed a significant decrease in the parasympathetic heart rate variability values during ‘head-up tilt’ indicating a decreased vagal modulation of heart rate in HD patients. These findings might suggest an over-stimulation of sympathetic activity and might be linked to an increased risk for syncopes and an increased overall cardiovascular risk [17].

The accumulation of intracellular pre-amyloid oligomers and higher assemblies has been observed in a number of human heart failure samples of various etiologies [18]. In a model of desmin-related cardiomyopathy produced by the cardiomyocyte-specific transgene expression of mutant αB-crystallin (CryAB^R120G^), aggregates were observed within cardiomyocytes leading to altered cardiomyocyte functions, perturbations in mitochondrial-sarcomere architecture, and deficits in mitochondrial function, which resulted in apoptosis and heart failure [19]. Moreover, a pro-amyloid potency of atrial natriuretic peptide (ANP) is often linked to isolated atrial amyloidosis and is presumed to be associated with congestive heart failure [20]. Recently, an artificial transgenic mouse model expressing either a mutant polyQ peptide of 83 glutamines (PQ83) or a control peptide of 19 glutamines (PQ19) under the control of the α-myosin heavy chain promoter (MyHC) to drive cardiomyocyte-specific expression has been reported [21]. PQ83 hearts developed cardiac dysfunction and dilation with a concomitant increase in the formation of insoluble aggregates in cardiomyocytes, and the mice invariably died before reaching eight months of age. The PQ83-induced heart failure was due to cardiomyocyte loss and, although apoptotic indices were unchanged in the PQ83 hearts, ultrastructural analysis revealed increased autophagic and lysosomal content indicative of increased autophagy. PQ83 hearts also showed evidence of necrotic death including inflammatory cell content and sarcolemmal permeability [21]. In this study, we have investigated the molecular and pathological features of the cardiac dysfunction that develops with disease progression in mouse models of HD and found that this occurs in the absence of mutant HTT deposits in the heart.

## Results

To test the hypothesis that mutant HTT leads to heart failure through compensatory remodelling as a consequence of cellular dysfunction, we used two well-established HD mouse models. R6/2 mice are transgenic for a mutated N-terminal exon 1 HTT fragment [22], while the *Hdh*Q150 mice have an expanded CAG repeat knocked-in to the mouse huntingtin gene (*Htt*) [23], [24], which is partially mis-spliced with the result that these mice express mutant versions of both an exon 1 HTT and a full length HTT protein [25]. For R6/2 mice, we studied cardiac function at all stages of disease: presymptomatic (4 weeks), symptomatic (8 weeks and 12 weeks) and end-stage (15 weeks). *Hdh*Q150 homozygotes were compared to wild type (WT) at 8 months of age (presymptomatic) and 22 months (end-stage disease).

**Figure 1:**
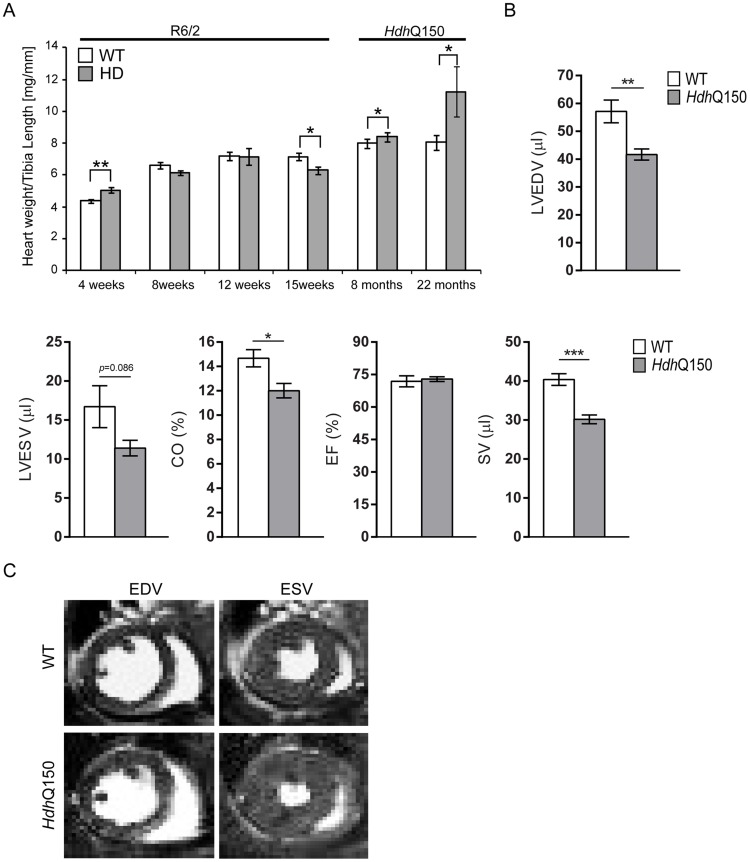
Morphometric and MRI analysis of HD mouse model hearts. (A) Heart weight to tibia length index (B) Comparison of the left ventricle end-diastolic volume (LVEDV), left ventricle end-systolic volume (LVESV), ejection fraction (EF) cardiac output (CO) and stroke volume (SV) between WT and *Hdh*Q150 mice at 22 months of age. There are significant differences in the LVEDV, LVESV, SV and CO. (C) End diastolic and systolic frames of a short axis myocardium slice for WT and *Hdh*Q150 mice. All values are mean ± SEM (*n* = 4/gender/genotype/age). Student's *t* test: *p<0.05, **p<0.01, ***p<0.001.

We began by quantifying the change in heart weight with disease progression (Fig. S1A). Given that both mouse models exhibit a decrease in body weight with disease progression, we opted to normalise heart weight to tibia length, a parameter that remains relatively constant between WT and HD mice during the course of disease (Fig. S1B). There was a significant increase in the heart weight to tibia index at 4 weeks of age in R6/2 mice (Fig. 1A) which reverted to a reduction by 15 weeks (Fig. 1A). In contrast the *Hdh*Q150 knock-in model showed an elevation of heart weight to tibia index at both 8 and 22 months of age (Fig. 1A). It has previously been reported that symptomatic R6/2 mice have abnormal cardiac function as measured by MRI at 12 weeks of age [26]. Therefore, we employed cardiac MRI to evaluate and compare the morphology and function of hearts from 22 month old *Hdh*Q150 mice. We found that the stroke volume (SV) and left ventricular end-diastolic volume (LVEDV) were significantly decreased, whereas the left ventricular end-systolic volume (LVESV) showed a decremental trend which was not statistically significant. (Fig. 1B and C). Consequently, cardiac output (CO) was also reduced, while ejection fraction (EF), due to its mathematical dependency on LVEDV and SV, remained unchanged (Fig. 1B and C).

**Figure 2:**
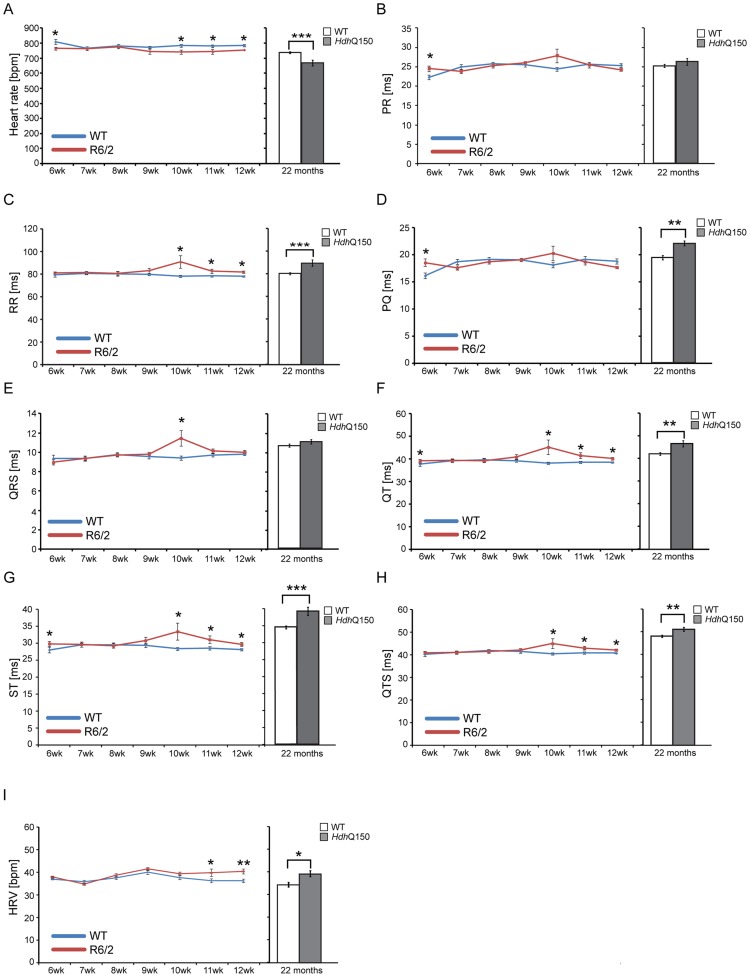
Resting ECG measurements for R6/2 and *Hdh*Q150 mice. ECGs recorded for R6/2 or *Hdh*Q150 mice and their respective WT littermates revealed a significant physiological difference in the symptomatic animals. (A) Heart rate, (B) PR interval, (C) RR interval, (D) PQ interval, (E) QRS interval (F) QT interval, (G) ST interval (H) QTS interval and (I) HRV. All values are expressed as mean±SEM (n≥6 mice per data point). All mice were conscious and unrestrained. One-way ANOVA with Bonferroni *post-hoc* test: *p<0.05, **p<0.01, ***p<0.001.

Next, to determine whether HD mice develop functional contractile abnormalities, we examined electrocardiogram (ECG) recordings in conscious mice between the HD mouse models and their respective WT littermates. We performed a longitudinal study in the R6/2 mouse model from 6 weeks of age (presymptomatic) to 12 weeks (symptomatic), whereas ECG parameters were only measured at 22 months of age for the *Hdh*Q150 mouse model. Heart rate was found to be significantly reduced at 10 weeks of age in R6/2 mice and at end stage disease in *Hdh*Q150 mice (Fig. 2A) which might suggest that symptomatic HD mice develop a moderate type of bradyarrhythmia that could progress to a cardiac arrest. The most common cause of bradyarrhythmia is heart block, detected through the elongation of the PR interval duration. However, surprisingly, the PR intervals were not significantly elongated in either HD model (Fig. 2B), although we did notice significantly elongated RR intervals (Fig. 2C). Also, we did not detect a progressive heart block manifesting in skipped beats with vanishing RR intervals. However, we did detect a significant elongation of PQ intervals in the *Hdh*Q150 while only trend towards elongation in R6/2 mice was found (Fig. 2D). Finally, we did not find any significant elongation of the time of ventricular depolarisation based on increased QRS intervals in either HD model (Fig. 2E). In contrast, the QT (Fig. 2F), ST (Fig. 2G) and QTS (Fig. 2H) intervals were already significantly elongated in the R6/2 mice by 10 weeks of age as well as in the end stage *Hdh*Q150 mice, which might be indicative of a long QT syndrome. In addition, the heart rate variability (HRV) was significantly higher in the symptomatic R6/2 mice at 11 and 12 weeks of age and in the *Hdh*Q150 mice at end-stage (Fig. 2I).

**Figure 3:**
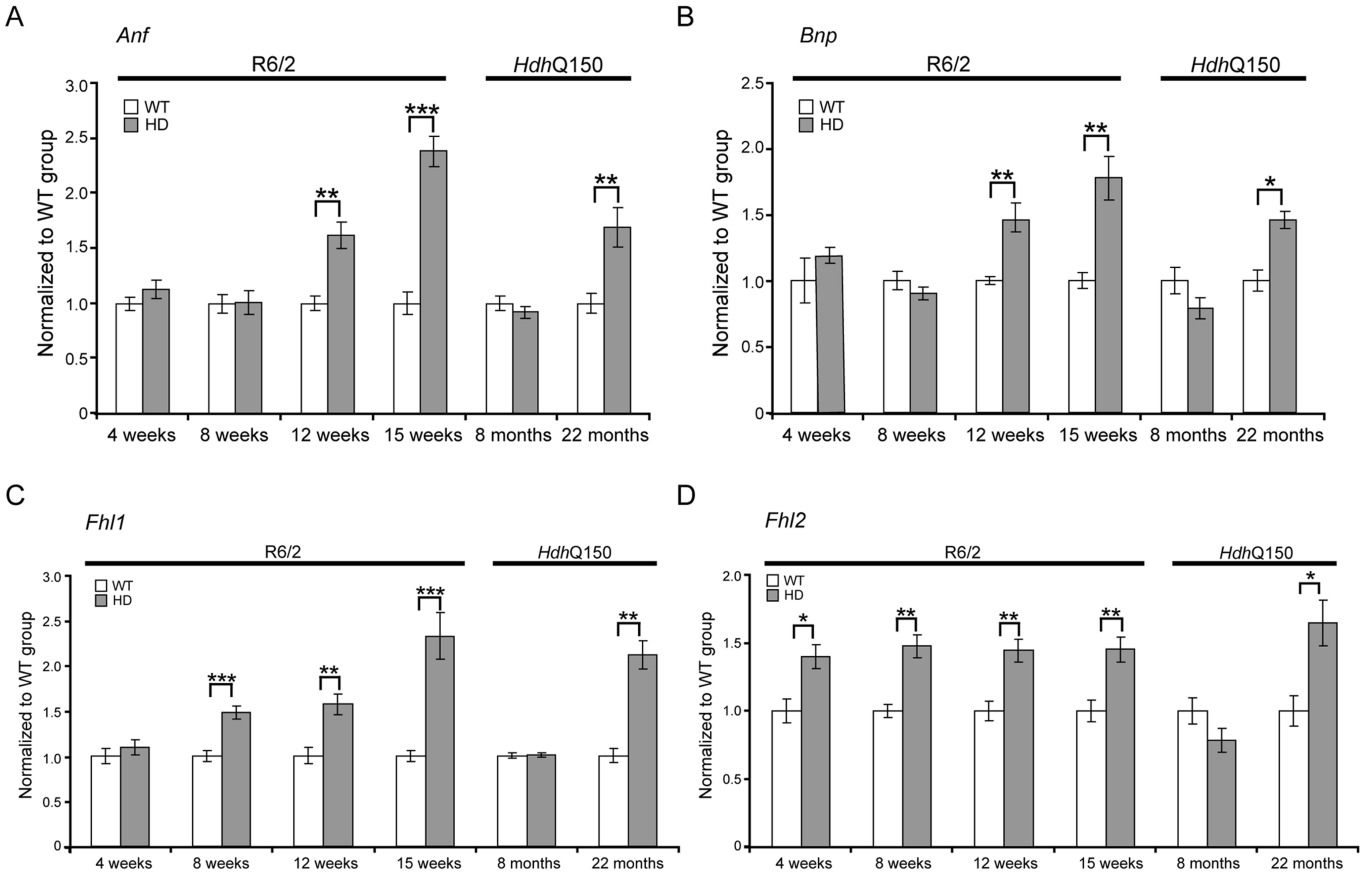
Activation of foetal gene markers in the hearts of R6/2 and *Hdh*Q150 mice. (A) *Anp* (atrial natriuretic peptide), (B) *Bnp* (brain natriuretic protein) and (C, D) members of the four and half LIM family *Fhl1* and *Fhl2 were* elevated in the heart of R6/2 and *Hdh*Q150 mice. All Taqman qPCR values were normalized to the geometric mean of three housekeeping genes: *Actb*, *Cyc1* and *Gapdh*. Error bars are SEM (n = 6). Student's *t*-test: *p<0.05, **p<0.01; ***p<0.001.

Pathological changes in the heart are often associated with the reactivation of a foetal gene programme and therefore, we assessed the expression levels of genes known to be changed as a consequence of hypertrophy or dilatated cardiomyopathy (DCM). Given that global transcriptional dysregulation is a pathogenic characteristic of HD, we first performed a systematic study to identify suitable reference genes for use in the expression analysis of hearts from HD mouse models. We used the geNorm Housekeeping Gene Selection Mouse Kit and associated software to identify the three most stably expressed genes in the hearts of symptomatic mice (Fig. S2). Our relative quantification methods then used the geometric mean of these three reference genes for normalization, to accurately determine gene expression levels in WT, R6/2 and *Hdh*Q150 mouse heart tissue. We found *Anp* (atrial natriuretic peptide) and *Bnp* (brain natriuretic peptide) to be up-regulated in symptomatic animals for both lines (Figs. 3A and B), but their expression level was unchanged at presymptomatic and early symptomatic stages. Two members of the four and half only LIM family, namely *Fhl1* and *Fhl2*, are also typically reactivated foetally-expressed genes. Both transcripts showed a significant up-regulation in R6/2 and *Hdh*Q150 murine hearts (Figs. 3C and D).

**Figure 4:**
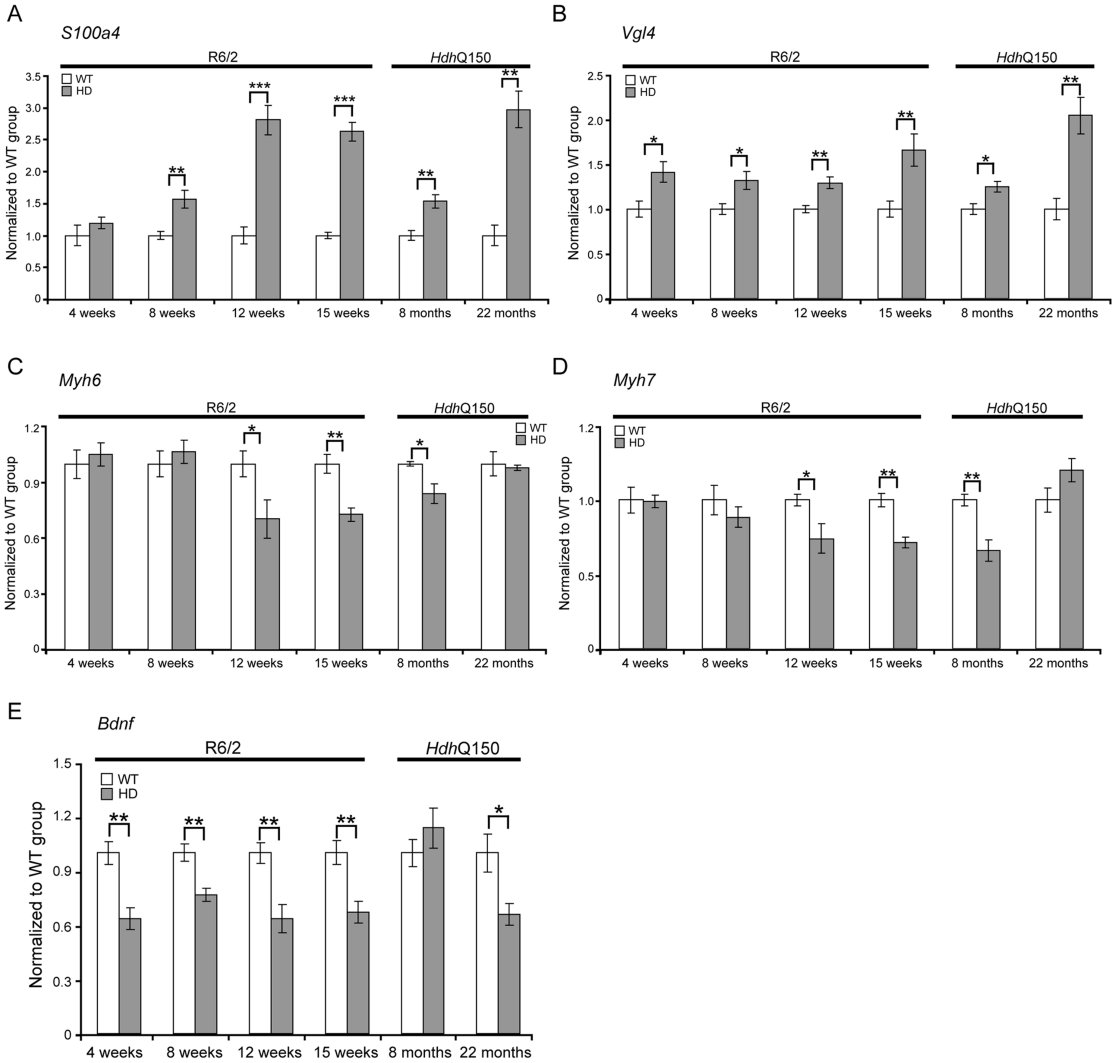
Transcriptional deregulation of gene markers involved in pathological processes. (A) *S100A4* (S100 calcium binding protein A4), (B) *Vgl-4* (vestigial related factor 4) transcripts were up-regulated while (C) *Myh6* (myosin heavy light chain 6) (D) *Myh7* (myosin heavy light chain 7) and (E) *Bdnf* (brain derived neurotophic factor) mRNAs were significantly decreased in the heart of R6/2 and *Hdh*Q150 mice. All Taqman qPCR values were normalized to the geometric mean of three housekeeping genes: *Actb*, *Cyc1* and *Gapdh*. Error bars are SEM (n = 6). Student's *t*-test: *p<0.05, **p<0.01; ***p<0.001.

To further examine the degree of heart pathology, we determined the expression levels of additional genes that are typically altered in diseased hearts. The multifunctional Ca2+-binding protein *S100A4* (also known as *Mts1* and *Fsp1*) is involved in fibrosis and tissue remodelling in several diseases including cancer, kidney fibrosis, central nervous system injury, and pulmonary vascular disease and has been shown to be increased in hypertrophic rat hearts [27]. We found that *S100A4* transcripts were significantly up-regulated in early-symptomatic R6/2 and presymptomatic *Hdh*Q150 hearts (Fig. 4A). *Vgl-4* (vestigial related factor 4) is a vital co-activator of the TEF (transcription enhancer family) and a marker of cardiac hypertrophy [28] and was already significantly up-regulated by 4 weeks of age in R6/2 mice and as early as 8 months in *Hdh*Q150 (Fig. 4B). Moreover, we also observed a significant reduction in the myosin heavy chain isoforms, *Myh6* and *Myh7* (Figs. 4C and D) in both mouse models, changes that are also indicative of foetal reprogramming. *Bdnf* (brain derived neutrophic factor) is down-regulated in the brain of HD mouse models [29], [30] and we found that its transcripts were also significantly down-regulated as early as 4 weeks of age in the hearts of R6/2 mice and at end-stage disease in *Hdh*Q150 mice (Fig. 4E).

**Figure 5:**
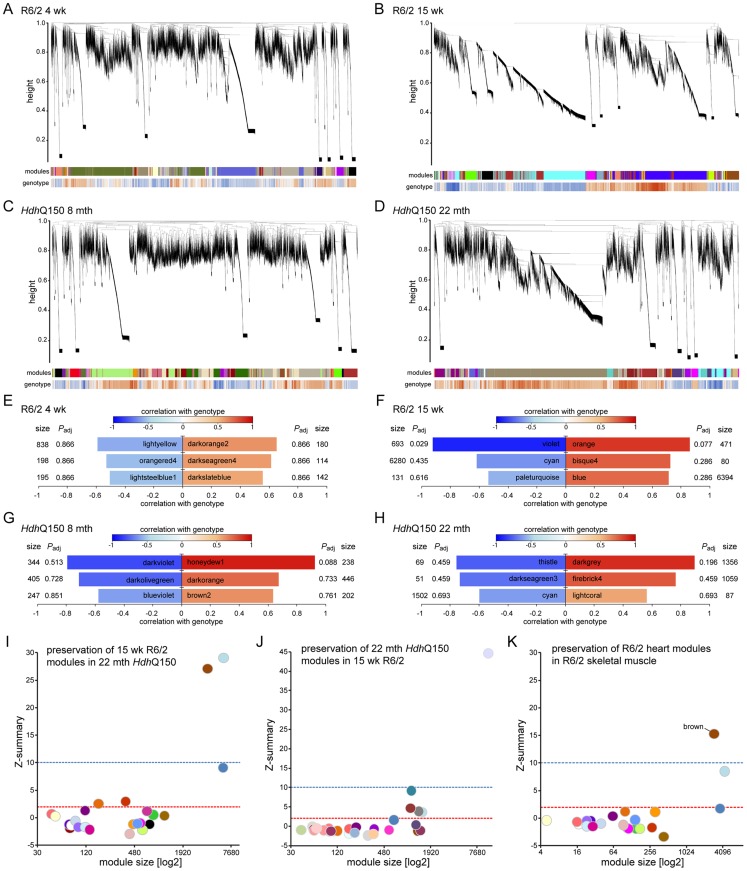
WGCNA analysis of heart RNAseq data. (A–D) Hierarchical cluster trees of the average linkage in the dissimilarity topological overlap matrices. Each vertical line correlates to a gene. The height is a measure for the dissimilarity based on the topological overlap. The first band under the dendrogram is a visual representation of the identified modules. The second band indicates the correlation with genotype based on the gene significance for each gene. Red is positively correlated with genotype, blue is negatively correlated. (A) Hierarchical cluster tree for WT versus R6/2 at 4 weeks of age. (B) Hierarchical cluster tree for WT versus R6/2 at 15 weeks of age. (C) Hierarchical cluster tree for WT versus *Hdh*Q150 at 8 months of age. (D) Hierarchical cluster tree for WT versus *Hdh*Q150 at 22 months of age. (E–H) Modules that were highly correlated with genotype in the respective networks are shown and are ranked according to their correlation with genotype. Size is the number of genes for each module. *P*adj gives the Benjamini Hochberg corrected significance value for each module. (E) Modules in the R6/2 network at 4 weeks of age. (F) Modules in the R6/2 network at 15 weeks of age. (G) Modules in the *Hdh*Q150 network at 8 months of age. (H) Modules in the *Hdh*Q150 network at 22 months of age. (I–K) Preservation analysis of late stage R6/2 and *Hdh*Q150 networks. The Z-summary is a measure for module preservation. Values less than 2 (red lines) indicate no preservation, between 2 and 10 (blue lines), module structures are preserved and above 10 the module structure is highly preserved. (I) Preservation of R6/2 modules at 15 weeks in the *Hdh*Q150 network at 22 months. (J) Preservation of *Hdh*Q150 modules at 22 months in the R6/2 network at 15 weeks. (K) Preservation of R6/2 modules at 15 weeks in a R6/2 network of skeletal muscle at 12 weeks.

As disease-related transcriptional dysregulation occurs in both the brain [31] and muscle [32] of both the R6/2 and *Hdh*Q150 HD mouse models, we applied RNAseq to document the extent of transcriptional changes in the hearts of R6/2 and *Hdh*Q150 mice at presymptomatic (4 weeks of age and at 8 months respectively) and symptomatic (12 weeks of age and 22 months respectively) stages. Differential expression analysis identified only one significantly dysregulated transcript in the R6/2 hearts at 4 weeks of age and very few significantly changed genes were identified in 8 month *Hdh*Q150 hearts (Table S1). Relatively few transcripts were dysregulated in the 22 month *Hdh*Q150 hearts (Table S1) and functional annotation clustering using DAVID failed to identify any significantly gene-ontology enriched clusters. The number of significantly altered transcripts was greater in 15 week R6/2 hearts (Table S1) including the myosin heavy chain genes: 6 and 7 that had already been assayed by qPCR. The functional gene-ontology clusters that were identified by DAVID to be significantly dysregulated included: the extracellular matrix, muscle proteins, the myosin complex, glycophosphatidylinositol-anchored proteins and immunoglobulins, consistent with a cardiac myopathy (Table S2). In order to determine whether we could identify genotype-specific patterns of dysregulation we applied weighted gene correlation network analysis (WGCNA). The hierarchical cluster trees for R6/2 at 4 and 15 weeks of age and *Hdh*Q150 at 8 and 22 months of age are illustrated in (Fig. 5A–D). This analysis only identified one module that significantly correlated with genotype and that was in the 15 week R6/2 hearts (Fig. 5F). This violet module was highly significantly enriched for genes encoding mitochondrial components, ribosomal proteins and those involved in glucose metabolism as well as genes encoding muscle and cardiac related proteins (Table S3). Preservation analyses were performed to compare module structure between these two models (Fig. 5I and J). None of the preserved modules for any of the comparisons correlated with the HD-genotype, as might have been expected at end-stage disease. Instead, the preservation analysis suggested that age is the major factor that determines the difference in transcriptional signatures between the two models. To determine whether the 15 week R6/2 heart transcriptome exhibited in HD-like signature we performed a preservation analysis against RNAseq of tibialis anterior muscle from R6/2 mice at 12 weeks of age. The large highly preserved module (brown) (Fig. 5K) was not correlated with genotype (Table S3), and further indicated that HD-related transcriptional dysregulation does not occur in cardiac tissue.

**Figure 6:**
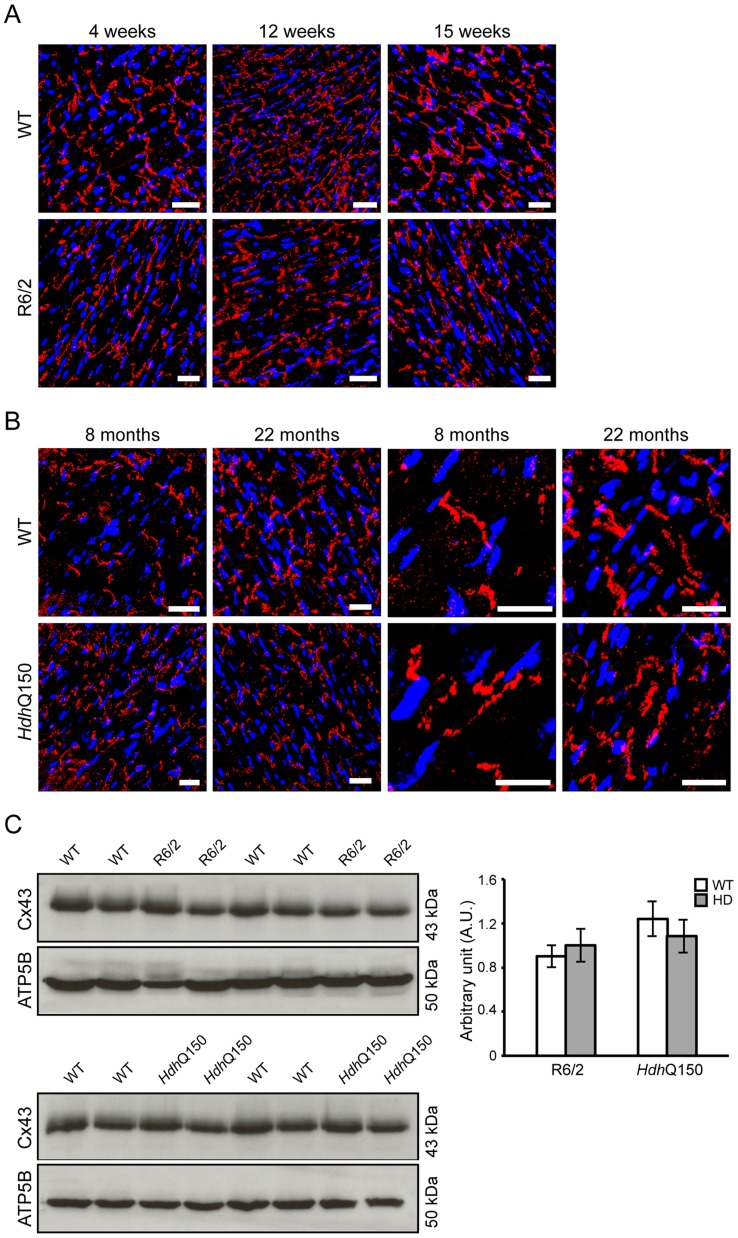
Altered connexin-43 localisation but not expression in the hearts of HD mouse models. Representative confocal pictograms of whole heart sections from (A) 4, 12 and 15 week old of R6/2 mice and (B) 8 and 22 month old *Hdh*Q150 mice showing low and high magnification. Anti-Cx-43 antibody (red) and nuclei (blue) were visualised with DAPI. Scale bar 30 μm. (C) Western blots of Cx-43 in 14-week-old WT and R6/2 mice (top panel) and in 22-month-old WT and *Hdh*Q150 mice (bottom panel). Relative expression of Cx-43 was normalized to ATP5b by densitometry and values are mean±SEM (*n* = 4). Student's *t* test: *p<0.05, **p<0.01, ***p<0.001.

It has been demonstrated that calcium overload may induce uncoupling of connexin43 (Cx43) from gap junctions and contribute to the occurrence of cardiac arrhythmia [33]. Gap junction remodelling, which is mainly formed by Cx43 in the ventricle, has been reported to contribute to an enhanced propensity for arrhythmogenesis [33]. In addition, the cardiac-specific knockout of Cx43 can lead to spontaneous ventricular arrhythmias with subsequent sudden cardiac death [34] and Cx43-deficient mice exhibit the accelerated onset and enhanced incidence of ventricular arrhythmias induced by ischemia [35]. Furthermore, it has been demonstrated that efferent vagal nerve stimulation protects the heart against ischemia-induced arrhythmias and that this is accompanied by prevention of the loss of phosphorylated Cx43 [36]. Therefore, Cx43 may serve as a marker of cardiac arrhythmias. We found that in HD mouse models Cx43 was significantly dislocated from the end plate towards the lateral membrane as early as 4 weeks of age in the R6/2 mice (Figs. 6A and Fig. S4) and 8 months of age in *Hdh*Q150 mice (Figs. 6B and Fig. S4). Surprisingly, we found that Cx43 was not changed at the protein level in either of these HD mouse models (Fig. 6C).

**Figure 7:**
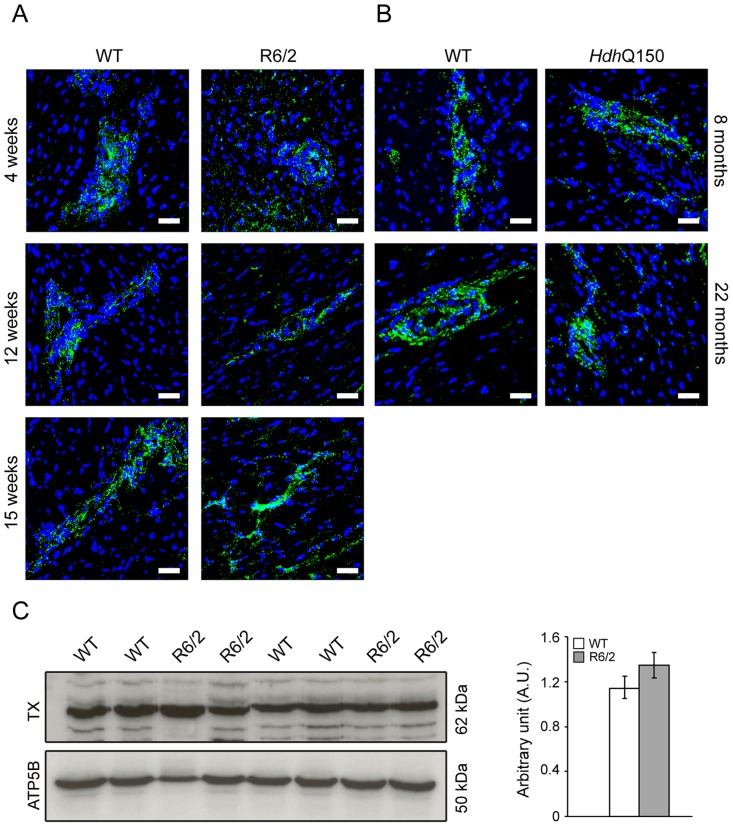
Architecture of altered ganglionic plexuses based on tyrosine hydroxylase localisation in HD mouse model hearts. Representative confocal pictograms of whole heart sections from (A) 4, 12 and 15 week old of R6/2 mice and (B) 8 and 22 month old *Hdh*Q150 mice. Anti-tyrosine hydroxylase antibody (green) was used to mark ganglionic plexuses and nuclei (blue) were visualised with DAPI. Scale bar 30 μm. (C) Western blots of tyrosine hydroxylase in 15-week-old WT and R6/2 mice. Relative expression of tyrosine hydroxylase was normalised to ATP5b by densitometry and values are mean ± SEM (*n* = 4). Student's *t* test: *p<0.05, **p<0.01, ***p<0.001.

There is evidence to suggest that the postganglionic sympathetic and intrinsic neurons in the heart are altered in Parkinson disease [37]. The majority of intrinsic cardiac ganglia are localized at the base of the heart at the roots of the pulmonary veins. These ganglia are interlinked by interganglionic nerves into the above mentioned nerve plexus of the heart hilum [38], [39]. In HD mouse models we found that the architecture of ganglionic plexuses was markedly altered in R6/2 mice by 12 and 15 weeks of age and in *Hdh*Q150 mice by 8 months (Figs. 7A and B) based on immunohistochemical labelling with tyrosine hydroxylase (TH). However, the expression level of TH was not changed in R6/2 mice at 15 weeks of age (Fig. 7C).

**Figure 8:**
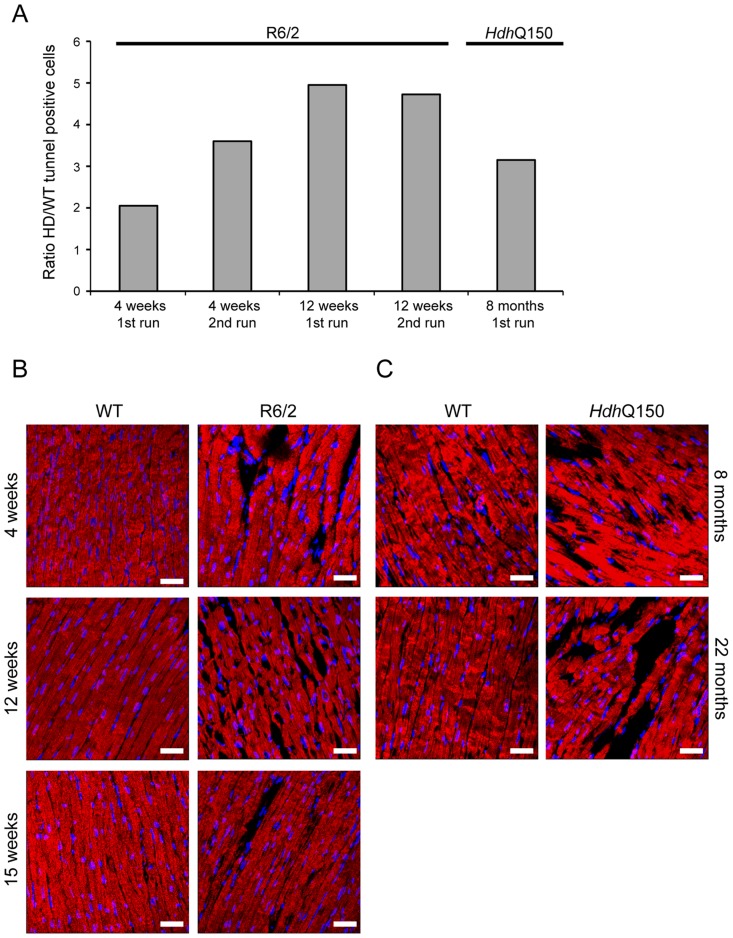
Ongoing cardiomyocyte death occurs through apoptosis in the hearts of HD mouse models. (A) TUNEL staining showed an increase of apoptotic activation in hearts from 4 and 12 weeks old R6/2 mice and 8 months old *Hdh*Q150 mice. The TUNEL assay was performed on 3 longitude sections from 3 animals (WT and HD) at each time point (for each of two separate runs). The fold change of apoptotic nuclei scored in HD animals (R6/2 or *Hdh*Q150) was normalized to their WT littermates. (B) Representative phalloidin staining (red) shows left ventricle myocyte degeneration and disarray in 4, 8 and 15 weeks old R6/2 mice. (C) Similar myocyte characteristics were found in the left ventricle myocytes from 8 and 22 months old *Hdh*Q150 mice. Nuclei (blue) were visualized with DAPI.

**Figure 9:**
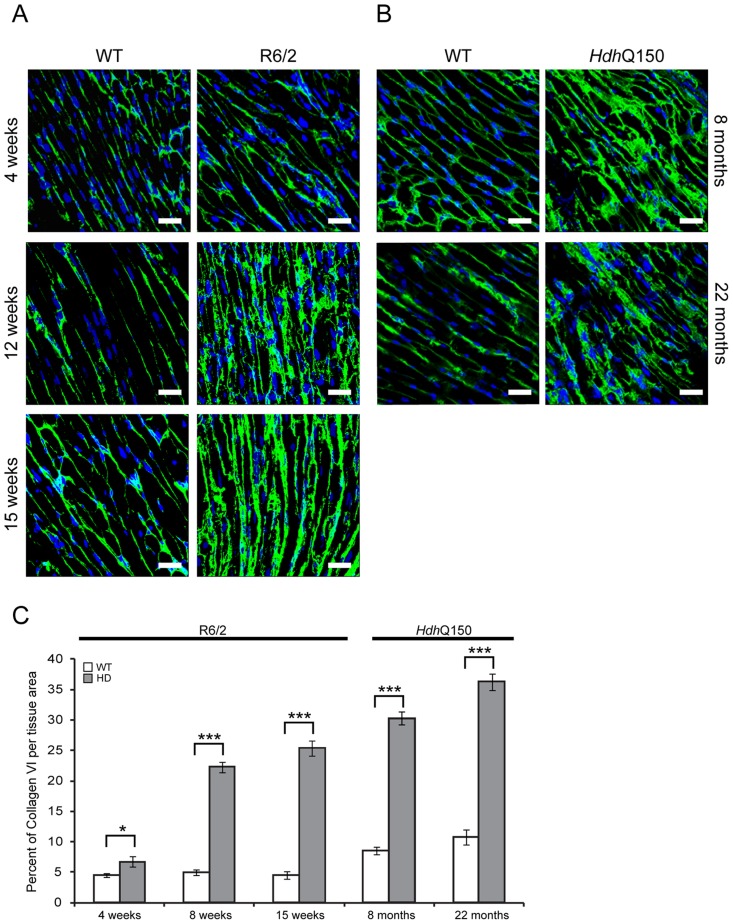
Minor to moderate fibrosis level based on collagen VI deposits in the hearts of HD mouse models. (A) Representative confocal pictograms of whole heart sections from 4, 12 and 15 week old R6/2 mice and (B) 8 and 22 month old *Hdh*Q150 mice. Fibrosis was detected with the anti-collagen VI antibody (green) and nuclei (blue) were visualised with DAPI. Scale bar 30 μm. (C) Quantification of the collagen VI staining area. Values are mean ± SEM (*n* = 4). Student's *t* test: *p<0.05, ***p<0.001.

In cardiomyopathic mouse models such as those that are transgenic for a mutant version of αβ-crystallin, amyloidosis is the source of the ongoing pathological features, including the activation of apoptosis in cardiomyocytes [18]. Apoptosis has been reported in the brain of an HD model [40]. To determine if this is also a common characteristic of HD mouse models, we used an immunofluorescence TUNEL assay to establish the degree of apoptosis in the hearts of both HD mouse models at different stages of disease. Cardiomyocytes from R6/2 mice displayed a significantly higher degree of apoptotic nuclei at 4 weeks of age as compared to WT littermates, which was further increased by 12 weeks (Fig. 8A and Fig. S3A). Similarly, *Hdh*Q150 mice showed increased numbers of cardiomyocyte apoptotic nuclei at 8 months of age (Fig. 8A and Fig. S3B). We were not able to obtain reliable data from the hearts of 22 month old *Hdh*Q150 mice due to the high background levels in their WT littermates. We conclude that cardiomyocytes die through an apoptotic-based process. The cardiomyocyte loss was also visualised by Phalloidin staining, by which the disarray and loss of cardiomyocytes was clearly visible (Fig. 8B and C). It is well established that cardiomyocyte death is accompanied by interstitial fibrosis. Therefore we used immunohistochemistry to detect the presence of collagen type VI deposits. This assay confirmed that the hearts of R6/2 mice develop a minor (4 weeks of age) to moderate (12 and 15 weeks of age) degree of fibrotic tissue (Fig. 9A and C) and we found a similar pattern of fibrotic labelling in the *Hdh*Q150 hearts (Fig. 9B and C). Therefore, we conclude that HD mouse models develop an interstitial type fibrosis and are free from the fibrotic patches that can be observed in desmin-related cardiomyopathy [41], [42].

**Figure 10:**
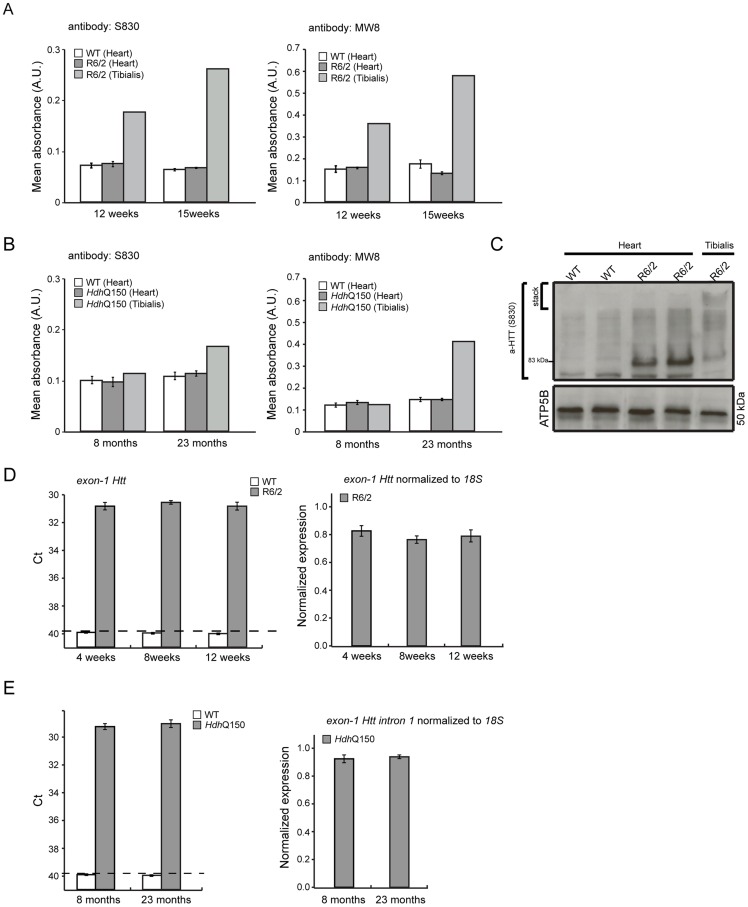
Mutant huntingtin aggregate species are not formed in the hearts of HD mouse models. (A, B) The seprion-ligand ELISA was used to identify and quantify aggregate loads in the heart lysates from R6/2 and *Hdh*Q150 mice. Error bars are S.E.M. (*n* = 6). (C) Representative S830 immunoblot of protein lysates showing the difference in soluble and aggregated exon-1 HTT between heart and skeletal muscle (tibialis anterior) of R6/2 mice at 14 weeks of age. ATP5b was used as loading control (lower panel). (D, E) Taqman qPCR showed that *HTT* exon-1 transgene levels are stable during the course of disease progression in the murine heart of R6/2 and *Hdh*Q150 mice. The dotted line indicates that the signal in WT animals occurs at the cut-off for gene expression. All Taqman qPCR values were normalized to the housekeeping gene 18S. Error bars are SEM (*n* = 6).

**Figure 11:**
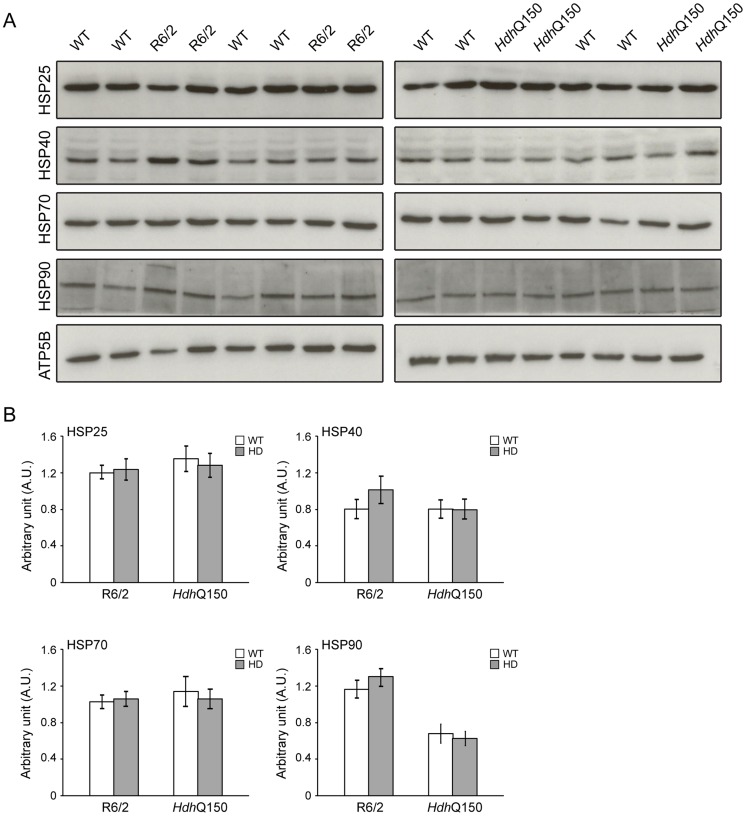
Heat shock proteins are not dysregulated in the murine HD hearts. (A) Western blots of heat shock proteins in 14-week-old WT and R6/2 mice (left panel) and in 22-months-old WT and *Hdh*Q150 mice (right panel). (B) Relative expression of HSP25, HSP40, HSP70 and HSP90 in 14-week-old WT and R6/2 mice and in 22-months-old WT and *Hdh*Q150 mice normalized to ATP5b by densitometry. Values are mean ± SEM (*n* = 4). Student's *t* test: *p<0.05, **p<0.01, ***p<0.001.

We have previously shown, that HTT inclusions can be detected throughout the periphery of the R6/2 and *Hdh*Q150 mouse models by immunohistochemistry [8], [9]. More recently, we have developed the seprion-ligand ELISA, a highly quantitative method with good statistical power that can be used to measure changes in aggregate load that occur *in vivo* in response to pharmacological or genetic manipulations [43]. Using this assay, we were unable to detect HTT aggregates in heart tissue from either R6/2 (Fig. 10A) or *Hdh*Q150 (Fig. 10B) mice, even at end stage disease. For R6/2 hearts, these data were supported by western blotting (Fig. 10C), which showed that the level of soluble exon 1 HTT was clearly visible and that aggregates had not been retained in the stacking gel (Fig. 10C). Consistent with these findings, although ubiquitin-positive deposits could be detected in the hearts of WT and *Hdh*Q150 mice at 22 months of age, and to a lesser extect in WT and R6/2 hearts at 12 weeks, these were not detected with the anti-HTT antibody S829 (Fig. S5). Next, we used Taqman qPCR to demonstrate that the absence of aggregates was not caused by a reduction in the exon-1 *HTT* mRNA in the heart of R6/2 (Fig. 10D) or *Hdh*Q150 (Fig. 10E) mice during the course of the disease. The basal levels of the major heat shock proteins: HSP40 and HSP70 become decreased in the brains of R6/2 mice with disease progression, potentially through the recruitment into HTT aggregates [44], [45]. By western blotting we could show that the levels of HSP40, HSP70, HSP25 and HSP90 were unchanged in the hearts of both R6/2 and *Hdh*Q150 mice at late-stage disease (Fig. 11) consistent with the lack of aggregate formation.

## Discussion

It is well established that amyloidosis comprises a large group of diseases characterised by the presence of misfolded proteins and their accumulation in the central nervous system, as well as in peripheral tissues such as heart and skeletal muscle. A number of studies have clearly identified amyloidosis as the pathological event leading to severe heart dysfunction in various mouse models and in humans [46]. Proof of concept studies in mice and flies have shown that the forced overexpression of an expanded polyQ track or mutant exon-1 HTT leads to heart failure and a significantly reduced lifespan for transgenic mice or flies that is associated with the deposition of aggregated protein [21], [47].

In this study we aimed to provide a broad spectrum of experimental insights into cardiac associated abnormalities that develop in the R6/2 transgenic and *Hdh*Q150 knock-in mouse models of HD, in which mutant *Htt* is expressed under the control of the *Htt* promoter. A cardiac phenotype had previously been noted in R6/2 mice using echocardiography and MRI [26], [48]. By using cardiac MRI in the *Hdh*Q150 mice, we found that all of the main volumetric parameters were decreased, thus highlighting a reduction in cardiac volumes as compared to WT. Cardiac output was also reduced, which might suggest that blood circulation had decreased while ejection fraction remained at normal levels. Such data are therefore highlighting a worsening in cardiac functions which might be responsible for cardiovascular deficits. Although the heart mass was increased at the end stage in *Hdh*Q150 mice, this was not true for R6/2 mice, which could be either explained by a self limitation of the mutant HTT effects themselves or by activation of compensatory hypertrophic pathways. The magnitude of these physiological changes was accompanied in both HD models by reactivation of a foetal gene programme, including the up-regulation of *Anp*, *Bnp*, *Fhl1* and *Fhl2* and a reduction in the α- and β- isoforms of the myosin heavy chain, all typical markers of an induced cardiomyopathy. Although, a similar type of cardiomyopathy accompanied with muscle wasting was observed when FOXO3 was over-expressed [49], our RNAseq data suggested that the level of *Foxo3* was not increased in the HD murine hearts.

At the pathological level, the HD-related cardiomyopathy was accompanied by a moderate degree of interstitial fibrosis in both HD mouse models. We also found that *S100a4* transcripts (a marker for fibrosis on several organs) were significantly elevated in both R6/2 and *Hdh*Q150 mice. Interestingly, *S100a4* mRNA has been up-regulated in the hypertrophic myocardium of the Dahl-rat hypertensive heart disease model and further activated during the transition to heart failure [27], [50]. Therefore, its' up-regulation might be indicative of hypertrophic stimuli in the hearts of both HD mouse models. Deposits of fibrotic tissues are a consequence of cardiomyocyte death and we found that cardiomyocyte loss is likely due to increased apoptosis in both HD mouse models, which was apparent as early as 4 weeks of age in R6/2 mice. It should be noted that these findings are in contrast to the artificial cardiac specific polyQ–overexpression model [21], in which cardiomyocyte death was found to be caused by necrosis.

It is well established that psychiatric and neurological diseases are positively linked to cardiovascular disease and that CNS abnormalities have a great impact on the pathogenesis of cardiac dysfunction [51]. In this study, we found that cardiac arrhythmias and pathology are features of HD mouse models. ECG telemetry in conscious mice showed that both symptomatic R6/2 and *Hdh*Q150 mice suffer from bradyarrhythmia. The reduction in heart rate and alterations in other ECG intervals were indicative of cardiac contractile abnormalities and were comparable to the previous findings in the spinal muscular atrophy mouse model [52]. Although there have been only a limited number of studies performed in HD patients, these were consistent with the HD mouse models in that relative heart rate was 10% lower in pre-symptomatic and early-symptomatic patients than in control subjects, likely due to a 10.6% lower diastolic pressure in early-symptomatic patients [53]. Similar findings were published recently for the R6/1 mouse model of HD, which had unstable RR intervals that were reversed following atropine treatment, suggesting parasympathetic nervous activation. The mice developed brady- and tachyarrhythmias, including paroxysmal atrial fibrillation and cardiac sudden death [54]. However, in contrast to our data, R6/1 mice had a higher heart rate than WT littermates in young but not older R6/1 mice [54].

The cardiac contractile dysfunction might be explained by the Cx43 dislocation from the end plate towards the lateral membrane, and we show for the first time, that this can be observed as early as 4 weeks of age in the R6/2 mice and 8 months of age in *Hdh*Q150 mice, while its protein levels remained unchanged. The ventricular myocytes are extensively coupled by gap junctions to meet physiological demands. In general, many heart diseases caused by different aetiologies are associated with changes in the expression of connexins or their remodelling. Thus, alterations in the structure or function of gap junctions have been linked to conduction disturbances and arrhythmogenesis in many heart diseases [55]–[57]. Moreover, the altered architecture of ganglionic plexuses based on immunohistochemical labelling with tyrosine hydroxylase and lower levels of *Bdnf* mRNA in HD murine hearts might also significantly contribute to contractile dysfunction in HD mouse models. These changes are likely to be driven by profound autonomic nervous dysfunction associated with widespread pathology of the central nervous system in HD.

In the brains of the R6/2 and *Hdh*Q150 mouse models, the accumulation of toxic mutant HTT aggregates [24], [58], [59] and transcriptional dysregulation [60] are early pathogenic events. To our surprise, and although we have been able to detect HTT aggregates in many peripheral tissues in both of these models [8], [9], [43], we were not able to detect mutant HTT aggregates in the heart lysates in either model by the sensitive seprion ligand ELISA [43]. Similarly, we failed to identify aggregates by immunohistochemistry in murine HD hearts using a broad spectrum of specific antibodies. These results are in stark contrast to the pathological deposition of polyQ aggregates that occurred in the model in which a polyQ peptide was overexpressed in cardiac tissue [21]. Consistent with the absence of aggregated HTT in nuclei, we were unable to identify a HD-specific signature of transcriptional dysregulation by RNAseq analysis.

**Figure 12:**
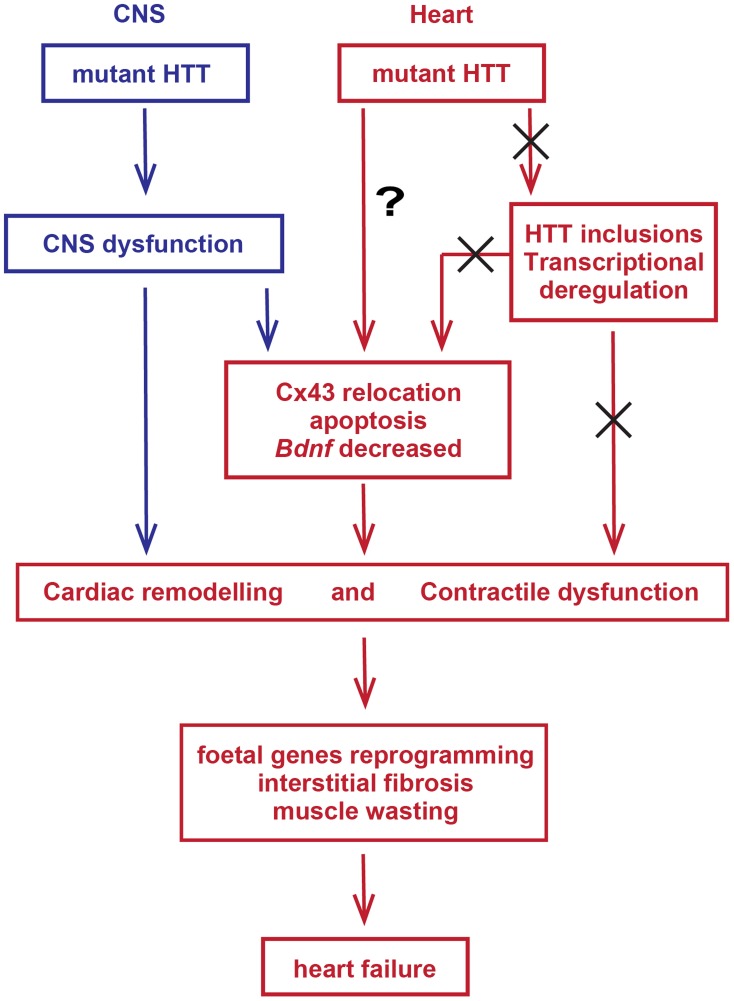
Model depicting the mechanism by which mutant huntingtin causes cardiac dysfunction in murine HD hearts.

In summary, mutant HTT results in the rapid development of pathological features that would be expected to lead to cardiac contractile dysfunction e.g. gap junction and ganglionic plexus remodelling and lower levels of *Bdnf* mRNA. In addition HD mouse models develop severe cardiac systolic and diastolic impairments likely due to ongoing cardiac remodelling represented by the re-expression of foetal genes and cardiomyocyte loss accompanied by a moderate level of interstitial fibrosis (Fig. 12). The increased heart rate variability, together with ganglionic plexus remodelling and the previously published pathological features in relevant brain regions in both mouse models [24], [54] would be consistent with a cardiac autonomic dysfunction. In HD patient brains, a recent meta-analysis of morphometric MRI found degenerative changes in the amygdala and insular cortex, even in the prodromal form on the disease [61]. Therefore, we postulate that HD related cardiomyopathy is likely driven indirectly by CNS dysfunction although it is not possible to rule out that intrinsic effects could contribute through a mechanism that has yet to be identified. At present it is not known whether cardiac dysfunction has clinical relevance for HD patients. There have been a number of functional studies, which have generally been performed on very small numbers of patients and have often been contradictory. Our finding, that cardiac dysfunction occurs in a genetically precise knock-in HD mouse model, suggests that there is an urgent need to perform well-designed unequivocal clinical studies to resolve this issue. It is important because of the potential liability for therapeutic intervention.

## Materials and Methods

### Ethics statement

All experimental procedures performed on mice were conducted under a project licence from the Home Office and approved by the King's College London Ethical Review Process Committee.

### Mouse maintenance and breeding

Hemizygous R6/2 mice were bred by backcrossing R6/2 males to (CBA x C57BL/6) F1 females (B6CBAF1/OlaHsd, Harlan Olac, Bicester, UK). *Hdh*Q150 homozygous mice on a (CBA x C57BL/6) F1 background were obtained by intercrossing *Hdh*Q150 heterozygous CBA/Ca and C57BL/6J congenic lines as described previously [24]. All animals had unlimited access to water and breeding chow (Special Diet Services, Witham, UK), and housing conditions and environmental enrichment were as previously described [62]. Mice were subject to a 12-h light/dark cycle. All experimental procedures were performed according to Home Office regulations.

### Genotyping

Genomic DNA was isolated from an ear-punch. R6/2 and *Hdh*Q150 mice were genotyped by PCR and the CAG repeat length was measured as previously described [43] and listed in Table S4. Dissected tissues were snap frozen in liquid nitrogen and stored at −80°C until further analysis.

### RNA extraction and Taqman real-time PCR expression analysis

Total RNA from whole hearts was extracted with the mini-RNA kit according to manufacturer instructions (Qiagen). The reverse transcription reaction (RT) was performed using MMLV superscript reverse transcriptase (Invitrogen) and random hexamers (Operon) as described elsewhere [30]. The final RT reaction was diluted 10-fold in nuclease free water (Sigma). All Taqman qPCR reactions were performed as described previously [63] using the Chromo4 Real-Time PCR Detector (BioRad). Stable housekeeping genes for qPCR profiling of hearts for HD mouse models were determined using the Primer Design *geNorm Housekeeping Gene Selection Mouse Kit with PerfectProbe*™ software. Estimation of mRNA copy number was determined in triplicate for each RNA sample by comparison to the geometric mean of three endogenous housekeeping genes (Primer Design) as described [30]. Primer and probe sets for genes of interest were purchased from Primer Design or ABI with the exception of *Anf*. The primers and Taqman probe for *Anf* were: Fw 5′>GAGCAAATCCTGTGTACAGTGCGG>3′, Rv: 5′>GCATCTTCTCCTCCAGGTGGTCTAG>3, probe: 5′>TCCAACACAGATCTGATG GATTTCAAG>3′ (Eurofins Operon) and the PCR product was verified by sequencing.

### RNA sequencing

Sequencing was performed by Expression Analysis on an Illumina Hi-seq 2000. Paired-end sequencing was obtained, 4-plexed across lanes for a minimum of 38 million 50mer paired reads per sample. Alignment and QC was conducted in Omicsoft using the OSA algorithm [64] against the mouse genome version B37. FPKMs were then calculated following standard formulas. QC assessment found all samples of high quality both at the RNA quality and alignment mapping levels. For each comparison (e.g. R6/2 vs WT or *Hdh*Q150 vs WT at specific ages), significance was assessed using DESeq [65] with 10% FDR and 30% fold change thresholds. The RNAseq data have been deposited in the GEO database under the accession number GSE58996.

### Weighted gene co-expression network analysis (WGCNA)

All networks were independently constructed from log2 transformed (FPKM+1) values of the heart RNAseq data. In principle, the workflow of the original publications was used [66]. Briefly, the pair wise weighted *Pearson* correlations between all pairs of genes across all samples were calculated. A signed adjacency matrix was calculated by raising the co-expression matrix to a soft-threshold power to reach approximate scale free topology of the network (R^2^>0.9). This power for the R6/2 4 week network was 30, 12 for R6/2 15 week, 26 for *Hdh*Q150 8 month and 28 for *Hdh*Q150 22 month. A signed topology overlay matrix was calculated based on the transformed connection strengths, which gives a biologically meaningful measurement of the similarity of the co-expression of two genes with all other genes in the network. Highly similar expressed genes were grouped by applying average linkage hierarchical clustering on the topology overlay matrix. Modules were subsequently identified by the dynamic hybrid tree cut algorithm [67]. Module eigengenes can be seen as representing the first principal component of a module. Modules with highly correlated module eigengenes were merged (*r*>0.7).

### Module preservation statistics

The WGCNA package includes statistical tests to analyse module preservation across different datasets [68]. Preservation is the similarity of interconnections between genes in a module, but also connectivity patterns of individual modules for the two data sets, i.e. high preservation is evidence for densely connected, distinct, and reproducible modules. We calculated 200 permutations of the preservation statistics and generated a Z-summary value by averaging them. The Z-summary indicates if a module is strongly preserved (Z-summary score >10), moderately preserved (Z-summary score 2<x<10) or not preserved (Z-summary score <2).

### Gene ontology analysis

Gene ontology analysis was carried out with the Database for Annotation, Visualization and Integrated Discovery (DAVID) Bioinformatics Resource [69]. We summarized all gene ontology terms (GO-term) of similar sub-terms into an overarching term. Benjamini-Hochberg corrected *p* values are shown for the respective GO-term.

## Antibodies, western blotting, Seprion ELISA

All primary and secondary antibodies used in this study are presented in Table S5. Preparation of protein lysates and western blotting were as described previously [63]. In general, 20 µg protein lysate was fractionated on 10% SDS-PAGE gels. Aggregates were captured in Seprion ligand coated plates (Microsens) and detected using the S830 sheep polyclonal or MW8 mouse monoclonal antibodies as described [43].

## Immunohistochemistry and confocal microscopy

For immunohistochemical studies, hearts were snap frozen in liquid nitrogen, or frozen in isopentane at −50°C, or incubated overnight in 4% PFA followed by overnight incubations in 20% and 30% sucrose in PBS, prior to embedding in OCT and storage at −80°C. 10–15 µm sections were cut using a cryostat (Bright instruments), air dried and immersed in 4% PFA in PBS or in acetone at −20°C for 15 min and washed for 3×5 min in 0.1% PBS-Triton X-100. Blocking was achieved by incubation with 5% BSA-C (Aurion) in 0.1% PBS-Triton X-100 for at least 30 min at RT. Immunolabeling with primary antibodies was performed in 0.1% PBS-Triton X-100, 1% BSA-C overnight in a humidity box at 4°C. Sections were washed 3x in PBS, incubated for 60 min at RT in a dark box with the anti-rabbit (FITC Invitrogen 1∶1000 in PBS), washed 3x in PBS and counterstained with DAPI (Invitrogen). Sections were mounted in Vectashield mounting medium (Vector Laboratories). Sections were examined using the Leica TCS SP4 laser scanning confocal microscope and analysed with Leica Application Suite (LAS) v5 (Leica Microsystems, Heidelberg, Germany). Quantification of the immunohistochemistry was performed using ImageJ (NIH).

## TUNEL assay

Terminal deoxynucleotidyl transferase (**T**dT) d**U**TP **N**ick-**E**nd **L**abeling (TUNEL) assay was used to detect apoptotic nuclei accordingly to the manufacturer's instruction (Roche). Sections were mounted in Vectashield mounting medium (Vector Laboratories) and examined using the Leica TCS SP4 laser scanning confocal microscope and analysed with Leica Application Suite (LAS) v5 (Leica Microsystems, Heidelberg, Germany).

## ECG evaluation in conscious mice

The ECG of conscious mice was recorded non-invasively using the ECGenie apparatus (Mouse Specifics, Inc., Boston, MA, USA). This device is a PowerLab-based system that acquires signal through disposable footpad electrodes located in the floor of a 6.5 cm by 7 cm recording platform. Several minutes of recording were collected for each mouse at weekly intervals from 4 to 14 weeks of age for R6/2 mice and at 8 and 22 months of age for *Hdh*Q150 mice. Briefly, segments of 2–3 s of recording (20–30 P-Q-R-S-T complexes) were chosen for analysis. Raw ECG signals were analysed using the eMOUSE software (Mouse Specifics). Heart rate was determined from the average of the RR interval, and short-term heart rate variability determination was based on the standard deviation of the RR intervals. Both parameters were expressed in beats per minute.

## MRI measurement

MRI was performed on a 7T horizontal MR scanner (Agilent) with mice in prone position as described [70], [71]. The gradient coil had an inner diameter of 12 cm, gradient strength was 1000 mT/m (100 G/cm), and rise-time 120 ms. Mice were scanned in a quadrature transmit/receive coil (RAPID Biomedical, Germany) with an internal diameter of 39 mm. Animals were initially anesthetized at 5% isoflurane, and then maintained at ∼1.5% isoflurane throughout the imaging procedure. Mice were kept warm using a warm air fan (SA Instruments, Stony Brook, NY). The ECG was monitored via two metallic needles placed subcutaneously in the front paws. A pressure-transducer for respiratory gating was placed on the animal abdomen.

Cine-FLASH MRI sequence was used to acquire temporally resolved dynamic short-axis images of the heart. Cine-FLASH was performed with ECG triggering only (single gating). Imaging parameters included TR = RR-interval/number of frames (typically 10 msec), TReff = RR-interval, TE = 1 msec, FOV = 20×25 mm, matrix size = 128×128, slice thickness = 1 mm; flip angle = 40 degrees, 3 averages, 9 slices, 1 k-space line/frame, 9 frames per cardiac cycle to study the dynamic contraction of the heart. The acquisition time was 8±0.5 minutes.

MRI images were analysed by the use of the ClinicalVolumes home-built segmentation analysis software (King's College London, www.clinicalvolumes.com) in order to obtain functional and volumetric information. Left ventricle end-diastolic volume (LVEDV), left ventricle end-systolic volume (LVESV), cardiac output (CO), Ejection fraction (EF), stroke volume (SV) were estimated [69].

## Statistical analysis

All data were analysed with Microsoft Office Excel and Student's *t*-test (two tailed) or ONE-WAY ANOVA with Bonferroni *post-hoc* test.

## Supporting Information

Figure S1 Morphometric analysis of HD mouse model hearts. (**A**) Heart weight and (**B**) Tibia length are shown. All values are mean ± SEM (*n* = 4). Student's *t* test: *p<0.05, **p<0.01, ***p<0.001.
(TIF)

Figure S2 Identification of suitable reference genes for qPCR from murine heart RNA from HD mouse models. A GeNorm analysis was used to identify optimal reference genes. Raw crossing threshold (C_t_) data for a panel of 12 potential references from the geNorm kit in wild-type and R6/2 mice at (A) 12 weeks and (B) 15 weeks of age. (C) A similar analysis was performed for 22 month old wild-type and *Hdh*Q150 mice. The following gene transcripts were examined: *Atcb* (Actin, beta, cytoplasmic, 11461), *Gapdh* (Glyceraldehydes-3-phosphate dehydrogenase, 14433), *Ubc* (Ubiquitin C, 22190), *B2m*, (Beta-2-microglobulin, 12010), *Ywhaz* (Phospholipase A2, 22631), *Rpl13a* (Ribosomal protein L13a, 22121), *Canx* (Calnexin, 12330), *Cyc1* (Cytochrome c-1, 66445), *Sdha* (Succinate dehydrogenase complex, subunit A, 66945), *18S* (18S rRNA, 19791), *Eif4A2* (Eukaryotic translation initiation factor 4A2, 13682), *Atp5b* (ATP synthase subunit, 11947).
(TIF)

Figure S3 Ongoing cardiomyocyte death occurs through apoptosis in the hearts of HD mouse models. (A) Representative pictogram of TUNEL staining in WT and R6/2 hearts at 4 and 12 weeks of age. (B) Representative pictogram of TUNEL staining in WT and *Hdh*Q150 hearts at 8 months of age. Arrowheads indicate apoptotic nuclei (red). Nuclei (blue) were visualized with DAPI. Scale bar 30 μm.
(TIF)

Figure S4 Quantification of the number of laterally localized Cx43 gap junctions. The number of laterally localized Cx43 labeled gap junctions per 100 nuclei (n = 3 mice/genotype). All values are mean ± SEM (*n* = 4). Student's *t* test: **p<0.01, ***p<0.001.
(TIF)

Figure S5 Accumulation of the ubiquitin positive deposits in the aged (22 months) but not younger (12 weeks) hearts. Representative confocal pictograms of whole heart sections from (A) 12 week old WT and R6/2 mice and 22 month old WT and *Hdh*Q150 mice. Anti-ubiquitin antibody (green), α-HTT (S830) (magenta) and nuclei (blue) were visualised with DAPI. Representative immunofluorescence images (B) of cortex from 12-week-old WT and R6/2 immunostained with α-HTT (S830) (magenta) antibody and DAPI (blue). Scale bar 30 μm.
(TIF)

Table S1 Significantly dysregulated transcripts as identified by differential expression analysis.
(XLSX)

Table S2 Gene ontology enrichment for differentially expressed gene between R6/2 and WT hearts at 15 weeks of age. Functional annotation was performed using the Database for Annotation, Visualization and Integrated Discovery (DAVID) Bioinformatics Resource (http://david.abcc.ncifcrf.gov/home.jsp). Hierarchical gene ontology (GO) terms were summarised into an overarching term. The enrichment score (overall importance) of the gene clusters and the corresponding Benjamini corrected *P*-value (*P*_adj_) are shown.
(DOCX)

Table S3 Gene ontology enrichment for highly correlated modules. Functional annotation was performed using the Database for Annotation, Visualization and Integrated Discovery (DAVID) Bioinformatics Resource (http://david.abcc.ncifcrf.gov/home.jsp). Hierarchical gene ontology (GO) terms for modules in the different networks were summarised into an overarching term. The enrichment score (overall importance) of the gene clusters and the corresponding Benjamini corrected *P*-value (*P*_adj_) are shown. A custom gene list with all genes in the respective network was used as background for the GO enrichment analysis. Only modules, for which a significantly associated GO-term (*P*_adj_<0.05) was found, are shown. For modules with more than 3000 genes, the 3000 genes with the highest absolute intramolecular connectivity (kME) were used for GO enrichment analysis.
(DOCX)

Table S4 Summary of the number of mice per genotype used in all studies and their CAG repeat sizes. SD = standard deviation.
(DOCX)

Table S5 Summary of the antibodies used in this study. Key: WB = Western Blotting; IHC = Immunohistochemistry; SEPRION = Seprion Ligand ELISA for aggregated huntingtin.
(DOCX)
