# Supplementary figures and images for: Dysfunction of the CNS-Heart Axis in Mouse Models of Huntington's Disease
Source: PLoS Genet. 2014 Aug 7;10(8):e1004550. doi: 10.1371/journal.pgen.1004550 (PMC4125112; doi:10.1371/journal.pgen.1004550)

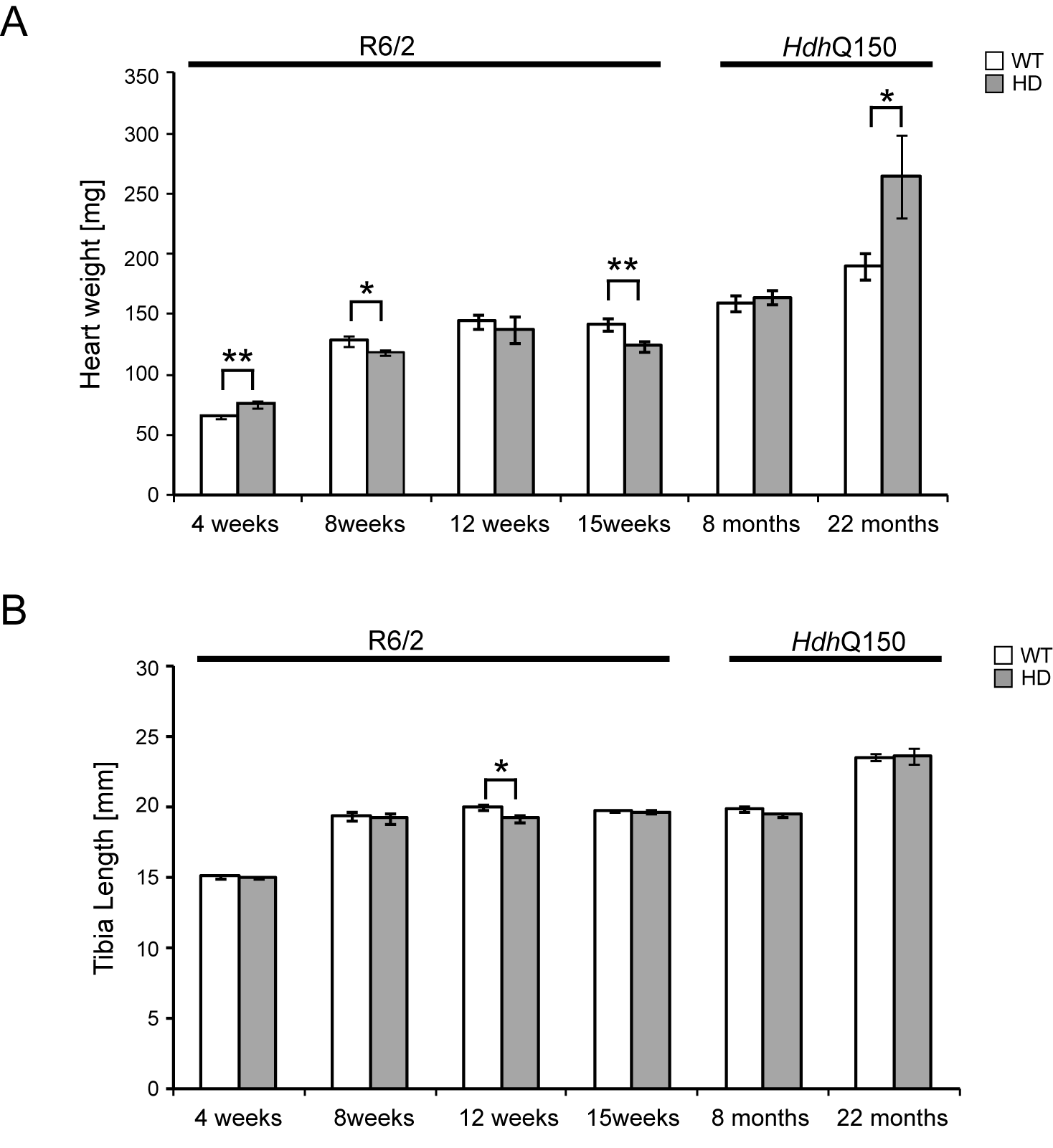

Supplement: Figure S1 — Morphometric analysis of HD mouse model hearts. (A) Heart weight and (B) Tibia length are shown. All values are mean ± SEM (n = 4). Student's t test: *p<0.05, **p<0.01, ***p<0.001. (TIF) [file pgen.1004550.s001.tif]

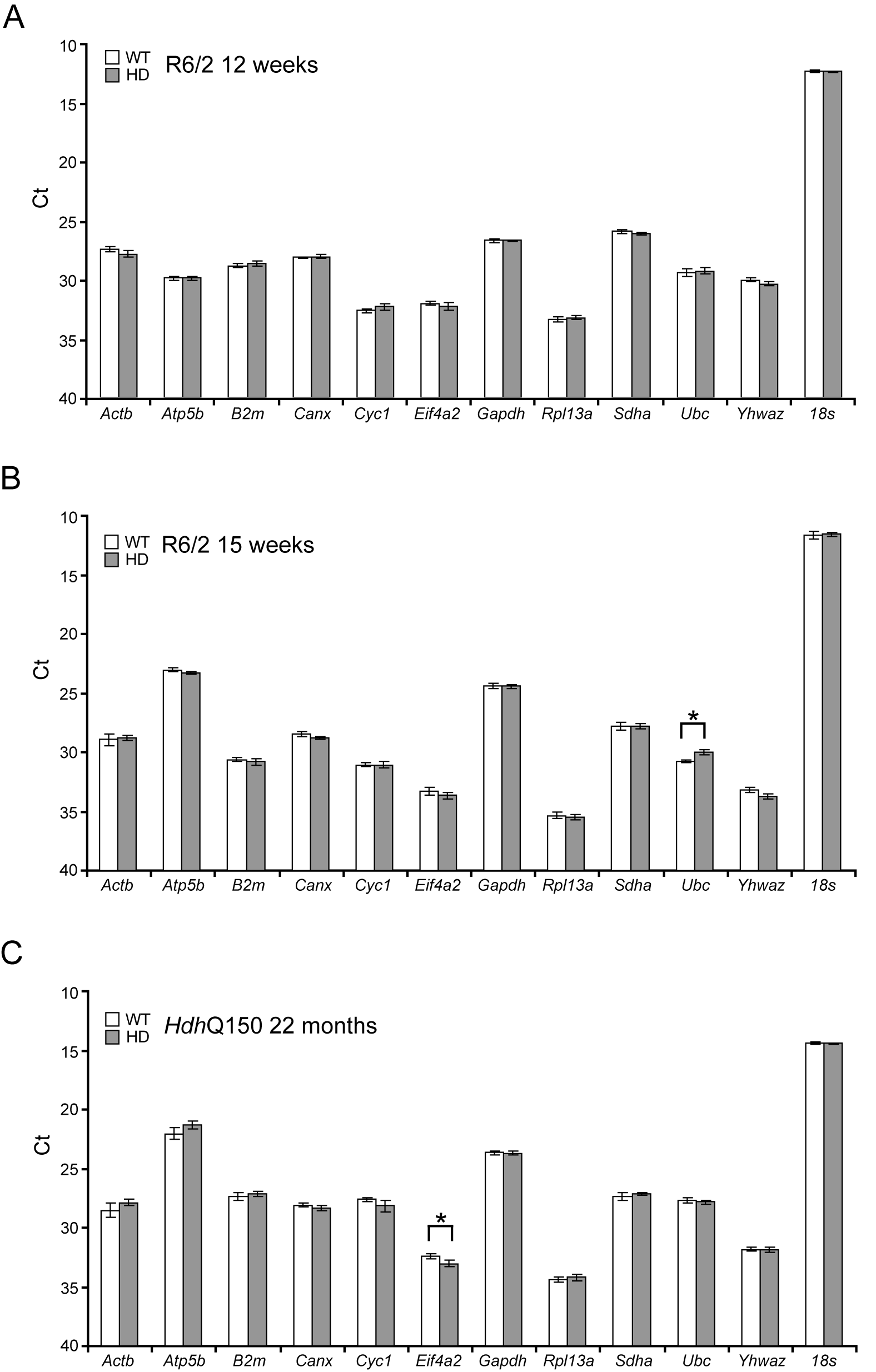

Supplement: Figure S2 — Identification of suitable reference genes for qPCR from murine heart RNA from HD mouse models. A GeNorm analysis was used to identify optimal reference genes. Raw crossing threshold (Ct) data for a panel of 12 potential references from the geNorm kit in wild-type and R6/2 mice at (A) 12 weeks and (B) 15 weeks of age. (C) A similar analysis was performed for 22 month old wild-type and HdhQ150 mice. The following gene transcripts were examined: Atcb (Actin, beta, cytoplasmic, 11461), Gapdh (Glyceraldehydes-3-phosphate dehydrogenase, 14433), Ubc (Ubiquitin C, 22190), B2m, (Beta-2-microglobulin, 12010), Ywhaz (Phospholipase A2, 22631), Rpl13a (Ribosomal protein L13a, 22121), Canx (Calnexin, 12330), Cyc1 (Cytochrome c-1, 66445), Sdha (Succinate dehydrogenase complex, subunit A, 66945), 18S (18S rRNA, 19791), Eif4A2 (Eukaryotic translation initiation factor 4A2, 13682), Atp5b (ATP synthase subunit, 11947). (TIF) [file pgen.1004550.s002.tif]

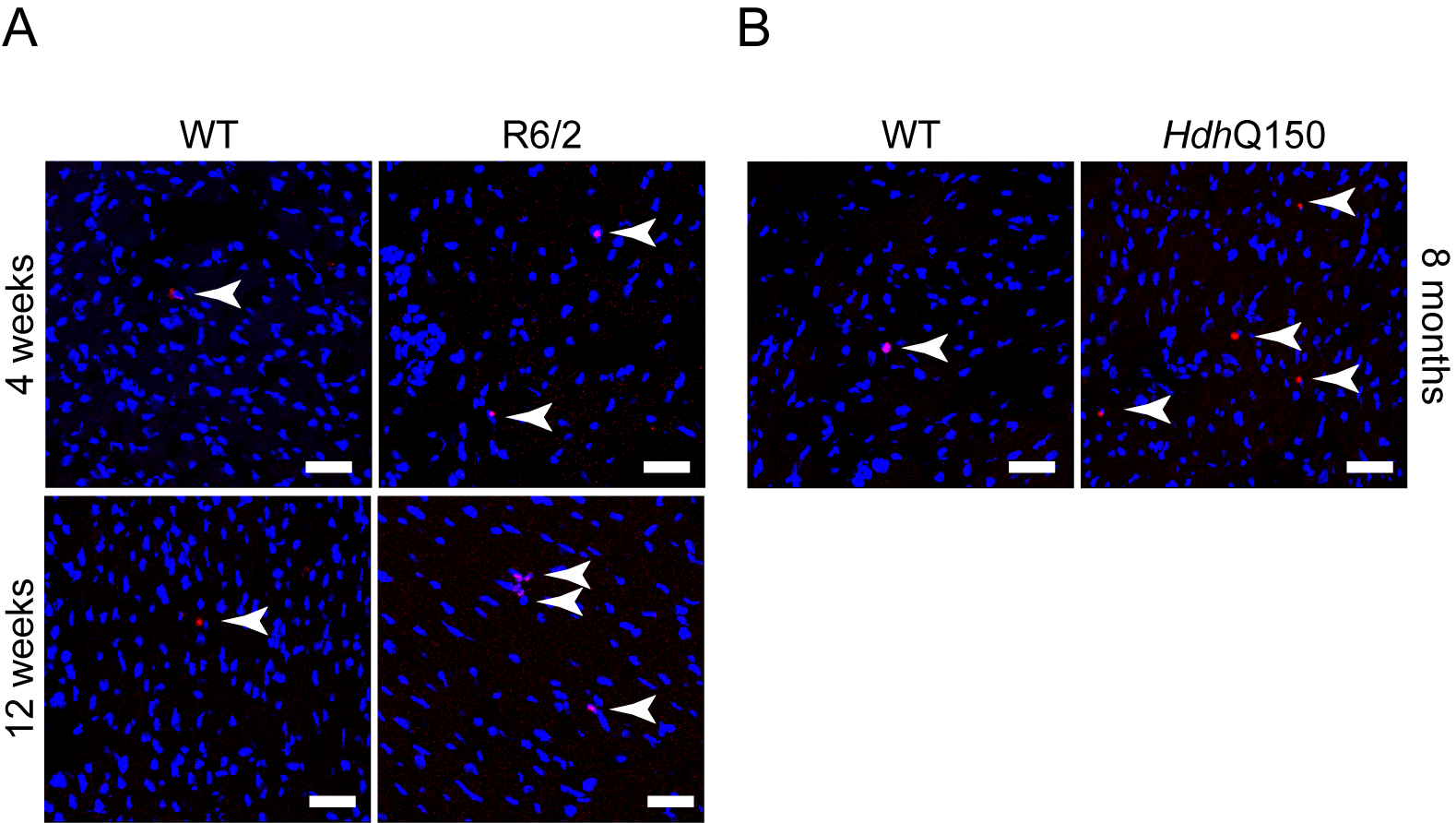

Supplement: Figure S3 — Ongoing cardiomyocyte death occurs through apoptosis in the hearts of HD mouse models. (A) Representative pictogram of TUNEL staining in WT and R6/2 hearts at 4 and 12 weeks of age. (B) Representative pictogram of TUNEL staining in WT and HdhQ150 hearts at 8 months of age. Arrowheads indicate apoptotic nuclei (red). Nuclei (blue) were visualized with DAPI. Scale bar 30 μm. (TIF) [file pgen.1004550.s003.tif]

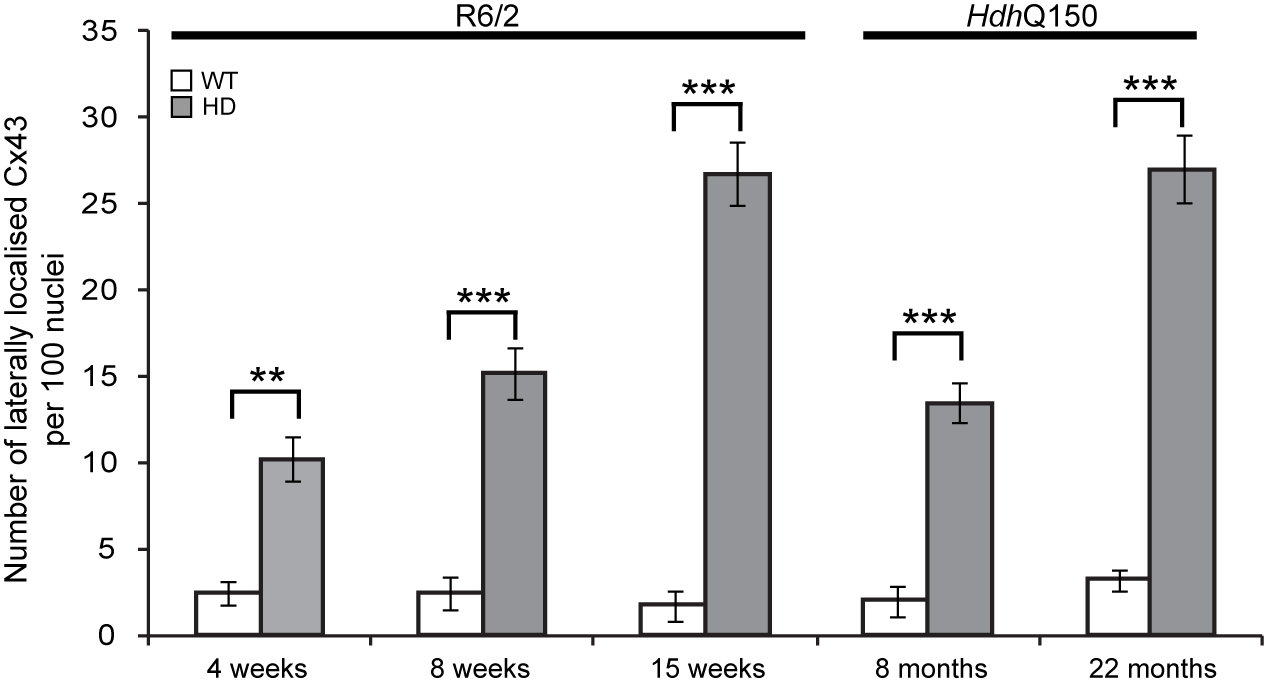

Supplement: Figure S4 — Quantification of the number of laterally localized Cx43 gap junctions. The number of laterally localized Cx43 labeled gap junctions per 100 nuclei (n = 3 mice/genotype). All values are mean ± SEM (n = 4). Student's t test: **p<0.01, ***p<0.001. (TIF) [file pgen.1004550.s004.tif]

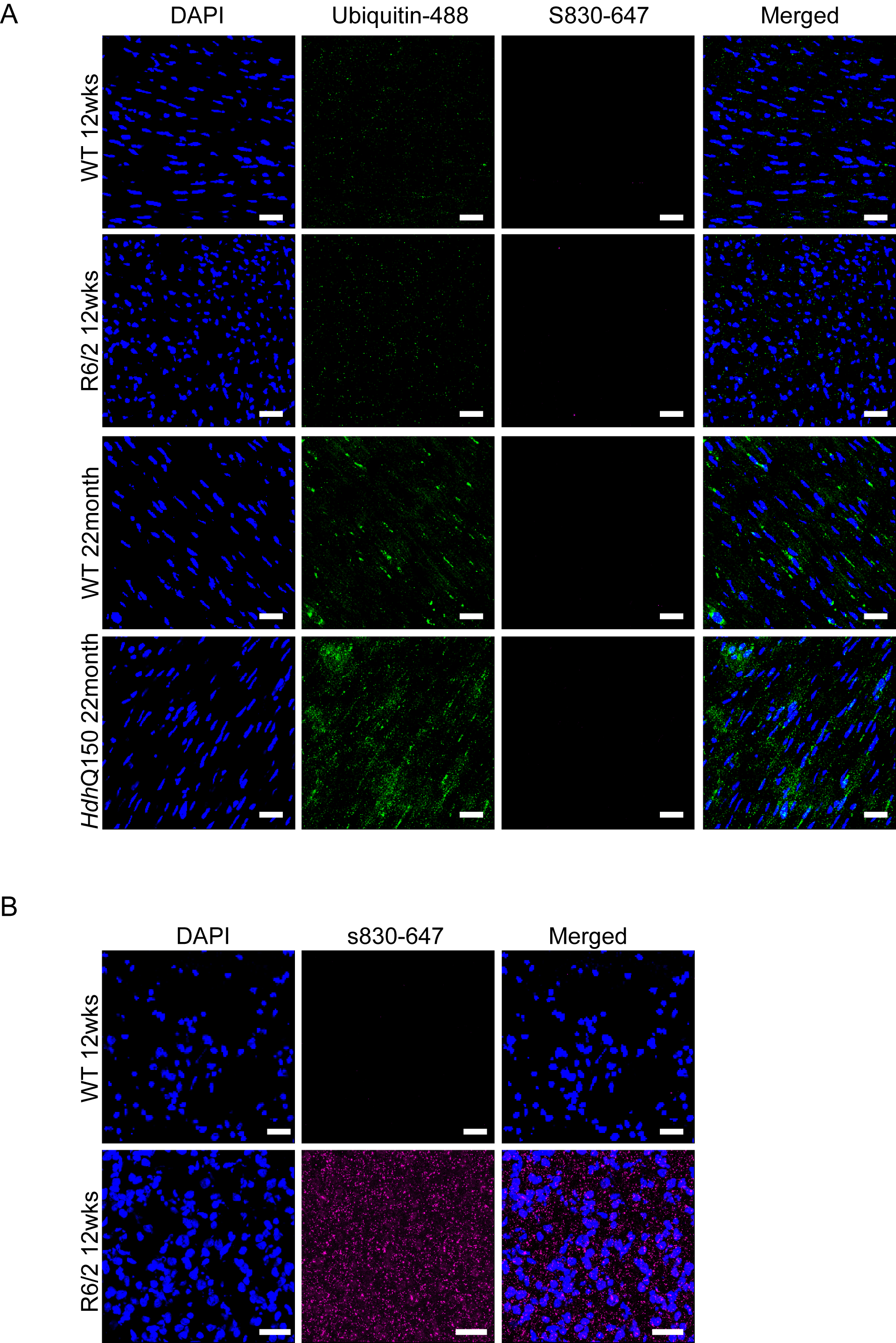

Supplement: Figure S5 — Accumulation of the ubiquitin positive deposits in the aged (22 months) but not younger (12 weeks) hearts. Representative confocal pictograms of whole heart sections from (A) 12 week old WT and R6/2 mice and 22 month old WT and HdhQ150 mice. Anti-ubiquitin antibody (green), α-HTT (S830) (magenta) and nuclei (blue) were visualised with DAPI. Representative immunofluorescence images (B) of cortex from 12-week-old WT and R6/2 immunostained with α-HTT (S830) (magenta) antibody and DAPI (blue). Scale bar 30 μm. (TIF) [file pgen.1004550.s005.tif]
