# Supplementary material for: Dysfunction of the CNS-Heart Axis in Mouse Models of Huntington's Disease
Source: PLoS Genet. 2014 Aug 7;10(8):e1004550. doi: 10.1371/journal.pgen.1004550 (PMC4125112; doi:10.1371/journal.pgen.1004550)
Supplement: Table S2 — Gene ontology enrichment for differentially expressed gene between R6/2 and WT hearts at 15 weeks of age. Functional annotation was performed using the Database for Annotation, Visualization and Integrated Discovery (DAVID) Bioinformatics Resource (http://david.abcc.ncifcrf.gov/home.jsp). Hierarchical gene ontology (GO) terms were summarised into an overarching term. The enrichment score (overall importance) of the gene clusters and the corresponding Benjamini corrected P-value (P adj) are shown. (DOCX) [file pgen.1004550.s007.docx]

| **GO-term (DAVID)** | **enrichment** | ***P*_adj_** |
| --- | --- | --- |
| **R6/2 - 15 week** | | |
| extracellular matrix | 5.41 | 3.7 ⋅10^-6^ |
| angiogenesis | 3.86 | 0.014 |
| circadian rhythm | 2.88 | 5.8 ⋅10^-4^ |
| muscle protein | 2.36 | 2.6 ⋅10^-4^ |
| immunoglobulin | 1.84 | 4.3 ⋅10^-4^ |
| contractile fiber | 1.61 | 0.032 |
| myosin complex | 1.50 | 0.009 |
|  |  |  |
