# Supplementary material for: Dysfunction of the CNS-Heart Axis in Mouse Models of Huntington's Disease
Source: PLoS Genet. 2014 Aug 7;10(8):e1004550. doi: 10.1371/journal.pgen.1004550 (PMC4125112; doi:10.1371/journal.pgen.1004550)
Supplement: Table S3 — Gene ontology enrichment for highly correlated modules. Functional annotation was performed using the Database for Annotation, Visualization and Integrated Discovery (DAVID) Bioinformatics Resource (http://david.abcc.ncifcrf.gov/home.jsp). Hierarchical gene ontology (GO) terms for modules in the different networks were summarised into an overarching term. The enrichment score (overall importance) of the gene clusters and the corresponding Benjamini corrected P-value (P adj) are shown. A custom gene list with all genes in the respective network was used as background for the GO enrichment analysis. Only modules, for which a significantly associated GO-term (P adj<0.05) was found, are shown. For modules with more than 3000 genes, the 3000 genes with the highest absolute intramolecular connectivity (kME) were used for GO enrichment analysis. (DOCX) [file pgen.1004550.s008.docx]

| **module** | **GO-term (DAVID)** | **enrichment** | ***P*_adj_** |
| --- | --- | --- | --- |
| **R6/2 - 4 week** | | | |
| darkseagreen4 | citrullination (chromatin) | 1.41 | 0.042 |
| lightyellow | DNA damage | 3.31 | 0.015 |
|  | zinc-finger transcription factors | 2.83 | 2.5 ⋅10^-3^ |
|  | interferon-inducible GTPase | 2.77 | 9.5 ⋅10^-8^ |
|  | protein transport | 1.86 | 0.017 |
| lightsteelblue1 | immune response | 1.73 | 0.037 |
| **R6/2 - 15 week** | | | |
| orange | mitochondrion  extracellular matrix | 10.54  2.33 | 5.0 ⋅10^-10^  0.012 |
| bisque4 | membrane proteins | 2.34 | 0.029 |
| blue | extracellular matrix | 14.9 | 3.2 ⋅10^-18^ |
|  | angiogenesis | 5.37 | 0.003 |
|  | membrane proteins | 5.15 | 1.1 ⋅10^-7^ |
|  | electron carrier activity | 2.87 | 0.014 |
|  | glutathione metabolism | 2.77 | 0.018 |
| violet | mitochondrion | 21.41 | 6.5 ⋅10^-30^ |
|  | ribosome | 5.55 | 7.6 ⋅10^-8^ |
|  | glycolysis / gluconeogenesis | 3.09 | 0.013 |
|  | muscle fibers | 1.13 | 0.038 |
| cyan | cell cycle | 15.97 | 1.3 ⋅10^-18^ |
|  | cytoskeleton | 15.41 | 1.3 ⋅10^-3^ |
|  | DNA damage | 9.55 | 1.7 ⋅10^-8^ |
|  | proteasome | 6.72 | 1.7 ⋅10^-6^ |
|  | ribosome biogenesis | 3.92 | 2.6 ⋅10^-5^ |
|  | lymphocyte differentiation / activation | 3.88 | 2.4 ⋅10^-3^ |
|  | mRNA processing | 3.86 | 1.3 ⋅10^-4^ |
|  | protein folding / chaperones | 3.72 | 8.4 ⋅10^-4^ |
|  | nuclear pore | 3.09 | 8.6 ⋅10^-3^ |
| brown | ribosome | 11.15 | 1.7 ⋅10^-21^ |
|  | regulation of transcription | 5.29 | 7.3 ⋅10^-6^ |
|  | mRNA processing | 4.44 | 3.3 ⋅10^-3^ |
|  | chromosome organization | 3.05 | 3.4 ⋅10^-4^ |
|  | protein transport | 2.55 | 3.1 ⋅10^-3^ |
| ***Hdh*Q150 - 8 month** | | | |
| darkviolet | mitochondrion | 10.9 | 9.5 ⋅10^-23^ |
|  | ribosome | 4.27 | 5.1 ⋅10^-7^ |
|  | glutathione metabolism | 2.83 | 2.4 ⋅10^-3^ |
| darkolivegreen | mitochondrion | 2.33 | 0.013 |
| ***Hdh*Q150 - 22 month** | | | |
| darkgrey | extracellular matrix | 6.77 | 1.3 ⋅10^-5^ |
|  | actin binding | 3.4 | 0.033 |
|  | GTPase activating | 2.46 | 0.048 |
|  | heart development | 1.98 | 0.016 |
|  | synaptosome | 1.97 | 0.041 |
| firebrick4 | regulation of transcription | 4.12 | 1.3 ⋅10^-6^ |
|  | angiogenesis | 3.57 | 0.028 |
| cyan | mitochondrion | 54.45 | 2.3 ⋅10^-102^ |
|  | ribosome | 6.07 | 2.2 ⋅10^-9^ |
|  | collagen / extracellular matrix | 4.59 | 1.1 ⋅10^-6^ |
|  | glycolysis / gluconeogenesis | 3.67 | 7.6 ⋅10^-4^ |
|  | lipid metabolism | 2.37 | 1.1 ⋅10^-3^ |
|  | heart development | 2.11 | 1.7 ⋅10^-3^ |
