# Supplementary material for: Dysfunction of the CNS-Heart Axis in Mouse Models of Huntington's Disease
Source: PLoS Genet. 2014 Aug 7;10(8):e1004550. doi: 10.1371/journal.pgen.1004550 (PMC4125112; doi:10.1371/journal.pgen.1004550)
Supplement: Table S4 — Summary of the number of mice per genotype used in all studies and their CAG repeat sizes. SD = standard deviation. (DOCX) [file pgen.1004550.s009.docx]

| Study | Genotype | Total number of mice | Mean CAG repeat Size | ±SD |
| --- | --- | --- | --- | --- |
| MRI | WT |  | - | - |
|  | *Hdh*Q150 |  | 160/180 | 4.2/9.5 |
| ECG | WT | 16 | - | - |
|  | R6/2 | 16 | 210 | 2.2 |
|  | WT | 14 | - | - |
|  | *Hdh*Q150 | 21 | 162/194 | 11.7/4.2 |
| Molecular biology tests  R6/2 and *Hdh*Q150 mice  Various studies: 4-14 weeks and 8 and 22 months respectively | WT | 50 | - | - |
|  | R6/2 | 50 | 204 | 2.5 |
|  | WT | 15 | - | - |
|  | *Hdh*Q150 | 18 | 165/192 | 10.5/5.2 |
