# Supplementary material for: Dysfunction of the CNS-Heart Axis in Mouse Models of Huntington's Disease
Source: PLoS Genet. 2014 Aug 7;10(8):e1004550. doi: 10.1371/journal.pgen.1004550 (PMC4125112; doi:10.1371/journal.pgen.1004550)
Supplement: Table S5 — Summary of the antibodies used in this study. Key: WB = Western Blotting; IHC = Immunohistochemistry; SEPRION = Seprion Ligand ELISA for aggregated huntingtin. (DOCX) [file pgen.1004550.s010.docx]

| **Antibody** | **Catalogue**  **number** | **Source** | **Dilution/**  **amount** | **Application** | **Reference** |
| --- | --- | --- | --- | --- | --- |
| S830 | N/A | In house | 1 in 1000 | WB | [[1](#_ENREF_1)] |
| S830 | N/A | In house | 1 in 1000 | IHC |  |
| S830 | N/A | In house | 1 in 2000 | Seprion |  |
| MW8 | N/A | P.Patterson | 1 in 4000 | Seprion | [[2](#_ENREF_2)] |
| HSP25 | SPA-801 | Stressgene | 1 in 1000 | WB | [45] |
| HSP40 | SPA-400 | Stressgene | 1 in 5000 | WB | [45] |
| HSP70 | SPA-810 | Stressgene | 1 in 1000 | WB | [45] |
| HSP90 | SPA-835 | Stressgene | 1 in 5000 | WB | [45] |
| ATP5b | AB14730 | Abcam | 1 in 30 000 | WB | N/A |
| Collagen VI | 600-401-108-0.1 | Rockland | 1 in 100 | IHC | N/A |
| Phloidine-Fitc | P5282 | Sigma | 1 in 100 | IHC | N/A |
| Phloidine-Tritc | P1951 | Sigma | 1 in 100 | IHC | N/A |
| Connexin 43 | MAB3067 | Millipore | 1 in 1000 | WB | N/A |
| Connexin 43 | C6219 | Sigma | 1 in 100 | IHC | N/A |
| Ubiquitin | Z045801-2 | Dako | 1 in 1000 | IHC | N/A |
| Tyrosine Hydroxylase | Ab6211 | Abcam | 1 in 1000 | WB | N/A |
| Tyrosine Hydroxylase | Ab6211 | Abcam | 1 :500 | IHC | N/A |
| α-Rabbit Alexa 555 | A21436 | Invitrogen | 1 in 1000 | IHC | N/A |
| α-Rabbit Alexa 488 | A21438 | Invitrogen | 1 in 1000 | IHC | N/A |
| α-Mouse Alexa 488 | A11001 | Invitrogen | 1 in 1000 | IHC | N/A |
| α-Rabbit Alexa 647 | A21245 | Invitrogen | 1 in 1000 | IHC | N/A |
| α-Goat Alexa 647 | A21447 | Invitrogen | 1 in 1000 | IHC | N/A |
| α-Mouse Alexa 488 | A21202 | Invitrogen | 1 in 1000 | IHC | N/A |
| Α-Rat HRP | P0450 | Dako | 1 in 3000 | WB | N/A |
| α-Goat HRP | P044901 | Dako | 1 in 3000 | WB | N/A |
| α-Mouse HRP | P0260 | Dako | 1 in 3000 | WB | N/A |
| α-Rabbit HRP | 32460 | Pierce | 1 in 20 000 | WB | N/A |
| α-sheep Alexa 488 | A11015 | Invitrogen | 1 in 1000 | IHC | N/A |
| α-sheep Alexa 555 | A21436 | Invitrogen | 1 in 1000 | IHC | N/A |

**REFERENCES**

1. Sathasivam K, Woodman B, Mahal A, Bertaux F, Wanker EE, et al. (2001) Centrosome disorganization in fibroblast cultures derived from R6/2 Huntington's disease (HD) transgenic mice and HD patients. Hum Mol Genet 10: 2425-2435.

2. Ko J, Ou S, Patterson PH (2001) New anti-huntingtin monoclonal antibodies: implications for huntingtin conformation and its binding proteins. Brain Res Bull 56: 319-329.
